# Inequality in Utilization of Maternal Healthcare Services in Low‑ and Middle‑Income Countries: A Scoping Review of the Literature

**DOI:** 10.1007/s10995-025-04111-9

**Published:** 2025-06-03

**Authors:** Farjana Misu, Dominic Gasbarro, Khurshid Alam

**Affiliations:** 1 Murdoch Business School, Murdoch University, Perth, WA 6150 Australia; 2 Department of Statistics, Jagannath University, Dhaka, 1100 Bangladesh

**Keywords:** Maternal healthcare, Inequality, Antenatal care, Postnatal care, Low- and middle-income countries

## Abstract

**Background:**

Inequality in maternal healthcare service (MHS) utilization is a significant global health challenge in low- and middle-income countries (LMICs). Recently, the literature on MHS inequality in LMICs has expanded. We conducted a scoping review to synthesize existing evidence and identify knowledge gaps.

**Methods:**

Following PRISMA-ScR guidelines, we systematically searched PubMed, Scopus, and CINAHL Ultimate in June 2023 for literature published since January 1, 2015. We included empirical studies using nationally representative data to measure inequality in at least one of five MHS indicators: antenatal care (ANC), skilled birth attendance (SBA), facility-based delivery (FBD), caesarean-section (C-section) delivery, and postnatal care (PNC). Our review encompassed 132 peer-reviewed articles on MHS inequality in LMICs.

**Results:**

ANC, FBD, and SBA were more frequently analyzed indicators for inequality measurement compared to PNC and C-section delivery. None of the 132 studies assessed all five MHS indicators together. The concentration index was the most frequently used inequality measure across all MHS indicators. Included studies were predominantly focused on economic (wealth) and geographic (residence, region) inequalities, while sociocultural factors (e.g., religion, ethnicity) remain underexplored. Inequality was most pronounced in low-income (LICs) and lower-middle-income countries (LwMICs). The extant literature mainly concentrates on India and Ethiopia as research settings.

**Conclusion:**

Our review highlights significant gaps in health inequality research, particularly in LICs and upper-middle-income countries (UMICs), with a heavy reliance on cross-sectional data, limited assessment of PNC and C-section delivery and lack of comprehensive analysis across all five common MHS indicators. Future research in LMICs should address the gaps identified in this review.

**Supplementary Information:**

The online version contains supplementary material available at 10.1007/s10995-025-04111-9.

## Significance

What is Already Known on this Study?

What this Study adds?

The use of maternal healthcare services (MHS) is vital for reducing mortality and morbidity rates. However, the utilization of MHS remains limited in low-and middle-income countries (LMICs), with discrepancies noted among different population groups.

Health inequality research in MHS is lacking in low-income countries, particularly in areas such as postnatal care and C-section delivery. Additionally, there is an insufficient comprehensive analysis across all five common MHS indicators. Sociocultural dimensions, such as religion and ethnicity, have also received insufficient attention. To address these gaps, future research in LMICs should focus on the areas identified in this review.

**Supplementary Information:**

The online version contains supplementary material available at 10.1007/s10995-025-04111-9.

## Background

Maternal health is recognized as one of the most critical challenges in global health (Masselos, 2021). This issue is especially pronounced in low- and middle-income countries (LMICs), which account for 95% of all maternal deaths in 2020 (WHO, 2020). Maternal healthcare encompasses a range of services, including antenatal care (ANC), skilled birth attendance (SBA), facility-based delivery (FBD), caesarean-section (C-section) delivery, and postnatal care (PNC) (Bobo et al., 2017; Pulok et al., 2018a, 2018b; Souza et al., 2024). ANC is critical for the early identification and management of high-risk pregnancies, significantly lowering the maternal mortality ratio (MMR) worldwide (Nita Ike Dwi Kurniasih, 2019; WHO, 2016). Following the antenatal period, FBD, SBA, and C-section delivery are linked to a substantial reduction in maternal and neonatal deaths (Shibre et al., 2020a; Tetteh et al., 2023; Yoseph et al., 2020). After delivery, timely access to PNC can reduce postpartum complications and prevent a substantial number of maternal deaths (Saldanha et al., 2023). Evidence suggests that nearly 80% of maternal mortality could be prevented if effective maternal healthcare were provided throughout pregnancy, delivery, and postpartum period (Prevention, 2022; Vale, 2023).

The utilization of quality maternal healthcare service (MHS) significantly influences the reduction of mortality and morbidity rates (Mekonnen et al., 2019; Reynolds et al., 2006). Hence, the UN Sustainable Development Goals (SDGs) emphasize maternal health by setting targets to lower the global MMR to 70 per 100,000 live births (Buse & Hawkes, 2015), and decrease neonatal mortality and mortality among children under five to below 12 and 25 per 1000 live births, respectively (Waeni, 2023). However, the use of quality MHS is limited in LMICs (Mangham-Jefferies et al., 2014) and is reported to have variations among population groups (Anindya et al., 2021). It can be argued that equality in the utilization of MHS is essential for improving maternal health and decreasing the MMR. SDG 3 is committed to promoting health and well-being for all ages, while SDG 10 aims to diminish inequalities and foster inclusivity and empowerment across and within nations (Tangcharoensathien et al., 2015). The literature identifies socio-cultural beliefs, geographic and financial inaccessibility, and environmental barriers to attaining equitable MHS in LMICs (Puchalski Ritchie et al., 2016).

A review article identified effective interventions for reducing maternal or child health inequalities in different sociodemographic groups in LMICs (Yuan et al., 2014). Another study reviews fundamental constraints in ANC by integrating various health services in different social and political contexts (Jongh et al., 2016). The impacts of continuity of care (COC) on both the mother and child's physical and mental health throughout the postnatal period have been extensively reviewed (D’haenens et al., 2020). A study used thematic analysis to identify geographic and socioeconomic factors influencing adolescent MHS in LMICs (Banke-Thomas et al., 2017). The progress of equity in MHS utilization from 2005 to 2015 was reviewed, emphasizing empirical analysis and the concentration index approach (Çalışkan et al., 2015). A qualitative summarization and meta-analysis assessed inequality in PNC services according to socioeconomic status and residence in LMICs (Langlois et al., 2015).

However, the existing review articles on MHS lack focus on inequality in all the common maternal health indicators, including ANC, SBA, FBD, C-section delivery, and PNC. Moreover, the number of studies on MHS, notably in the LMICs, has outpaced existing reviews, which are limited by time and scope. In particular, after the adoption of SDGs in 2015, LMIC literature on inequality in MHS has not been covered in the existing reviews. To bridge this gap, we set out to conduct a scoping review to consolidate recent evidence on inequality in MHS in the LMICs based on the World Bank classification of countries by income as of 2023 (i.e., low-income [LIC], lower-middle-income [LwMIC] and upper-middle-income countries [UMIC] with a gross national income per capita of US$ 13,845 or less). The overarching research questions steering this review are: How has inequality in MHS been assessed? And how is MHS unequally distributed among women when it is utilized? The specific objectives are to review the indicators of MHS utilization and methodologies, and equity strata used in the recent literature to measure inequality in the LMIC population, determine differences across country income groups, and identify the level of inequality for the most frequently assessed equity stratum across countries. So, our current review compiled a broader collection of recent studies on the inequality of household-level MHS in the LMICs in Africa, Asia, southeastern Europe, and Latin America, covering the years 2015 to 2023.

## Methods

**Fig. 1:**
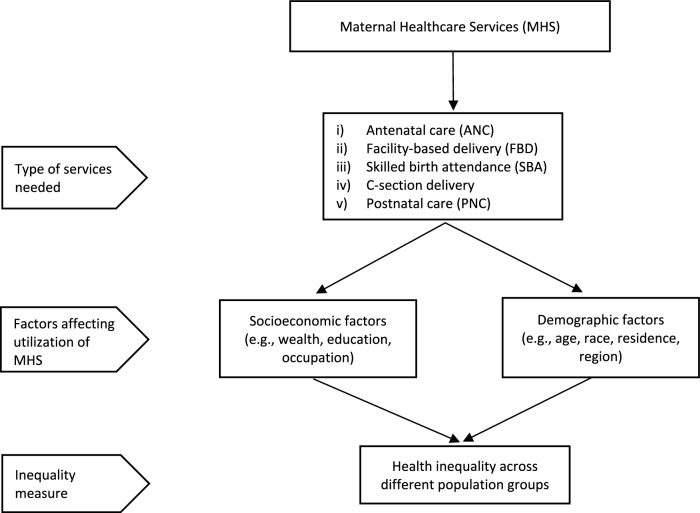
Conceptual framework of health inequality

We developed a conceptual framework (Fig. 1) on inequality in MHS based on existing literature to guide the scoping review. Health inequality encompasses various social and economic dimensions (McCartney et al., 2013), measured by variations in health status, outcomes, and healthcare experiences among different socio-demographic groups (Dawson et al., 2019). Structural theory suggests that disparities in socioeconomic conditions account for differences in health outcomes (McCartney et al., 2013), and reducing structural inequalities decreases health inequities (Kakama & Basaza, 2022). Figure 1 illustrates that socioeconomic and demographic factors influencing MHS utilization contribute to inequality among population groups. We consider ANC, SBA, FBD, C-section delivery, and PNC as MHS utilization indicators in this study to evaluate a country's MHS provision across all population groups, as using a smaller subset of indicators could yield inaccurate conclusions or create misleading incentives for policymakers.

### Search Strategies

With the support of Murdoch University's subject librarians, we developed a scoping review protocol, which was pilot-tested and calibrated before final data collection. We searched PubMed, Scopus, and CINAHL Ultimate for empirical literature on health inequality in maternal healthcare services (MHS) in low- and middle-income countries (LMICs). Our search was guided by a population, interest, context (PICo) statement that defined the research question, inclusion and exclusion criteria, and constructed the search terms. The search terms consisted of three blocks:"Maternal Healthcare Services,""Inequality,"and"Low- and middle-income countries,"with each block containing related synonyms. The search included the names of countries classified as low-income, lower-middle-income, and upper-middle-income by the World Bank in June 2023 (Bank). The searches carried out on 23 June 2023 employed time, language, and publication type filters to find peer-reviewed articles in English published since 2015. The search strings are detailed in Additional File 1.

### Study Selection

**Fig. 2:**
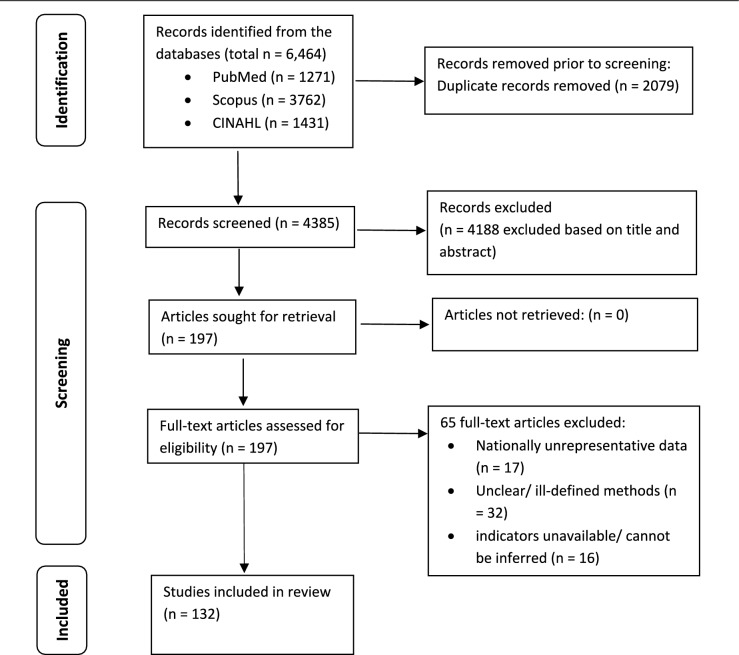
Flow chart of screening and selection processes

We adhered to the Preferred Reporting Items for Systematic Reviews and Meta-Analysis Extension for Scoping Reviews (PRISMA-ScR) guidelines for study selection (Tricco et al., 2018). Studies from the initial literature search were uploaded as CSV files into the Nested Knowledge Platform (Adusumilli et al., 2022) to eliminate duplicates and screen titles and abstracts for eligibility based on stringent inclusion and exclusion criteria. The inclusion criteria were original research articles in scholarly journals; studies on countries listed by the World Bank as LICs, LwMICs, and UMICs as of June 2023 (Bank); retrospective observational studies using nationally representative data; studies focusing on quantitative analysis of health inequality in MHS, assessing any of the five selected MHS indicators; and studies employing World Health Organization (WHO) (WHO, 2013) or World Bank (O'Donnell et al., 2008) recommended health inequality measures. Exclusion criteria included working papers, review articles, qualitative studies, and analyzes focusing solely on MHS utilization without explicit inequality measures. Reviewer (FM) initially screened titles and abstracts to assess inclusion or exclusion status. The selected studies were then reassessed for eligibility, and full texts were reviewed for suitability. A second reviewer (KA) confirmed the final selection based on the criteria. Figure 2 illustrates the study selection process.

### Health Inequality Measures

Inequality measures are summary metrics used to describe health differences between population subgroups, stratified by factors such as income, education, residence, sex, or ethnicity (WHO, 2013). Both WHO and the World Bank emphasize using a mix of simple and complex measures to monitor health inequalities. Simple measures (e.g., difference, ratio) compare two groups, typically the most and least advantaged (WHO, 2013). Complex measures (e.g., slope index of inequality, relative index of inequality, concentration index, population attributable fraction, Theil index, coefficient of variation) assess inequality across the entire population, considering all subgroups and their population sizes (WHO, 2013, 2021). These measures provide more nuanced interpretations—such as the average difference in health across the socioeconomic spectrum (slope index) or how health outcomes are distributed relative to population rank (concentration index). The World Bank also applies decomposition analyzes (e.g., concentration index decomposition, Blinder-Oaxaca, and Fairlie methods) to identify the contribution of factors like wealth and education to observed inequalities (O'Donnell et al., 2008). For the visual presentation of inequalities, WHO recommends tools like Equiplots and concentration curves. The concentration curve plots the cumulative health variable against the cumulative population ranked by socioeconomic status, with greater deviation from the equality line indicating greater inequality (WHO, 2013). A detailed explanation and interpretation of inequality measures, along with examples, are provided in Additional File 1.

### Data Extraction and Synthesis

The selected studies were analyzed based on geographical focus (countries studied), data sources and types, assessed MHS indicators and measures (as recommended by WHO or World Bank) used to determine MHS inequality. We examined inequality levels in MHS using prevalent measures and predictors. Studies focused on any of the five MHS indicators (ANC, SBA, FBD, C-section, and PNC) were explored. For ANC, we included studies measuring indicators such as ANC in the first trimester, early ANC, any ANC, ANC by a skilled provider, and various ANC visit counts (1 +, 3 +, 4 +, etc.), as well as quality ANC and full ANC. For PNC, we considered studies examining indicators for women or newborns, including any PNC, 1 + PNC visits, multiple PNC visits, PNC within 24 h, within 2 months, adequate PNC, within 2 weeks, within 6 weeks, 3 + PNC visits within 6 weeks, PNC before discharge, 4 + PNC visits, within 2 days/48 h, and within 40 days.

## Results

### Study Selection

After screening 4385 titles and abstracts, we reviewed 197 full-text peer-reviewed articles and included 132 in the study. The main exclusion reasons were (1) unavailability or non-inferable MHS indicators (16 studies used composite indexes instead of individual MHS indicators); (2) unclear or poorly defined methods or concepts (32 studies did not report MHS indicator inequality based on WHO or World Bank recommended measures); and (3) MHS analysis from non-nationally representative data (17 studies). Approximately 40% of the included studies were published between 2021 and mid-2023, with around 68% published between 2019 and mid-2023 (Table 1).

**Table 1:** Distribution of the selected studies by geographical coverage and data sources used

Panel A: Single-country studies (n = 104)				
Country	Income group, region^a^	Studies reviewed	Percentage of studies reviewed (N)	Data source
India	LwMIC, SA	(Godha & Hotchkiss, 2022); (Gandhi et al., 2022); (Yadav & Jena, 2020); (Krishnamoorthy et al., 2020); (Sk et al., 2022); (Chauhan & Jungari, 2021); (Ali et al., 2020); (Gandhi et al., 2021); (Kumar et al., 2019); (Vellakkal et al., 2017); (Himanshu & Källestål, 2017); (Panda et al., 2020a); (Panda et al., 2020b); (Guilmoto & Dumont, 2019); (Chauhan & Radkar, 2023); (Mishra et al., 2021); (Ali et al., 2021); (Paul, 2021); (Shirisha et al., 2022)	18.3 (19)	National Family Health Surveys 1992/93, 1998/99, 2005/06, 2015/16, 2019/21; NSSO unit-level data 1995, 2004, 2014; Public Affairs Index 2016; District Level Household and Facility Surveys 1995/99, 2000/04, 2006/07, 2007/08, 2011/12, 2012/13; Annual Health Survey 2011/12
Ethiopia	LIC, SSA	(Tsegaye et al., 2022); (Gebre et al., 2018); (Mezmur et al., 2017); (Memirie et al., 2016); (Shibre et al., 2020a); (Shibre et al., 2023); (Tesfaye et al., 2017); (Ambel et al., 2017); (Onarheim et al., 2015); (Daka et al., 2020); (Bobo et al., 2017); (Tarekegn et al., 2022)	11.5 (12)	DHS 2000, 2005, 2011, 2014, 2016, 2019
Ghana	LwMIC, SSA	(Seidu et al., 2022); (Novignon et al., 2019); (Okyere et al., 2022); (Kpodotsi et al., 2021); (Asamoah & Agardh, 2017); (Amporfu & Grépin, 2019); (Abekah-Nkrumah, 2018); (Dankwah et al., 2019); (Tetteh et al., 2023); (Anarwat et al., 2021); (Ekholuenetale et al., 2021)	10.6 (11)	DHS 1993–2014; MICS 2011; Malaria Indicator Survey 2019
Bangladesh	LwMIC, SA	(Pulok et al., 2018a); (Rahman et al., 2017a, 2017b); (Rahman et al., 2022a); (Kamal et al., 2016); (Pulok et al., 2016); (Rahman, et al., 2022b); (Pulok et al., 2020); (Chowdhury et al., 2023); (Khan et al., 2018); (Anwar et al., 2015)	9.6 (10)	DHS 1993/1994, 1996/1997, 1999/2000, 2004, 2007, 2011, 2014, 2017/18; Maternal Mortality and Health Care Surveys 2001, 2010
Nepal	LwMIC, SA	(Pandey et al., 2021); (Shreezal & Adhikari, 2023); (Bhusal, 2021); (Mehata et al., 2017); (Thapa et al., 2020); (Sapkota et al., 2021); (Ali et al., 2023)	6.7 (7)	DHS 1996, 2001, 2006, 2011, 2016; MICS 2019; Family Health Survey 1996
Nigeria	LwMIC, SSA	(Okoli et al., 2020); (Nwosu & Ataguba, 2019); (Agho et al., 2016); (Adeyanju et al., 2017); (Ushie et al., 2019); (Fagbamigbe & Oyedele, 2022)	5.8 (6)	DHS 1990, 2003, 2008, 2013, 2018
Brazil	UMIC, LAC	(França et al., 2016); (Mallmann et al., 2018); (Silva et al., 2018); (Flores et al., 2021); (Fonseca et al., 2022)	4.8 (5)	DHS: 1986, 1996, 2006; National Health Survey 2013; Live Births Information System data 2000–2015, 2020
Tanzania	LwMIC, SSA	(Bintabara & Basinda, 2021); (Bintabara, 2021); (Shibre et al., 2020b)	2.9 (3)	DHS 1996, 1999, 2004, 2010, 2015/2016
Vietnam	LwMIC, EAP	(Lam et al., 2019); (Kien et al., 2019); (Nguyen et al., 2021)	2.9 (3)	MICS2000, 2006, 2011, 2014; DHS 2002
Ecuador	UMIC, LAC	(Rios Quituizaca et al., 2021); (Quizhpe et al., 2020); (Rios-Quituizaca et al., 2022)	2.9 (3)	Reproductive Health Surveys 1994, 1999, 2004; National Survey of Health and Nutrition 2012; LSMS 2006, 2014
China	UMIC, EAP	(Liang et al., 2017); (Fan et al., 2017)	1.9 (2)	National Health Service Surveys 2008, 2013; National Maternal and Child Health Annual Report office 2000‐2013; National Health Statistics Yearbook 2014; China Women and Children Statistical Information 2014;
Indonesia	UMIC, EAP	(Nababan et al., 2017); (Zahroh et al., 2020)	1.9 (2)	DHS 1991, 1994, 1997, 2002/3, 2007, 2012, 2017
Philippines	LwMIC, EAP	(Hodge et al., 2016); (Paredes, 2016)	1.9 (2)	DHS 2008, 2013
Afghanistan	LIC, SA	(Akseer et al., 2016); (Kim et al., 2016)	1.9 (2)	MICS 2010/2011; Mortality Survey 2010
Sierra Leone	LIC, SSA	(Tsawe & Susuman, 2022); (Jalloh et al., 2019)	1.9 (2)	DHS 2008, 2013, 2019
Mauritania	LwMIC, SSA	(Shibre et al., 2021); (Taleb El Hassen et al., 2022)	1.9 (2)	MICS 2007, 2011, 2015
Zimbabwe	LwMIC, SSA	(Lukwa et al., 2022)	1.0 (1)	DHS 2015
South Africa	UMIC, SSA	(Wabiri et al., 2016)	1.0 (1)	National HIV Prevalence, Incidence, Behaviour and Communication Surveys 2008, 2012
Burundi	LIC, SSA	(Yaya et al., 2020)	1.0 (1)	DHS 2010, 2016
Togo	LIC, SSA	(Atake, 2021)	1.0 (1)	DHS 1998, 2013
Kenya	LwMIC, SSA	(Keats et al., 2018)	1.0 (1)	DHS 2003, 2008, 2014
Iran, Islamic Rep	LwMIC, MENA	(Bayati et al., 2017)	1.0 (1)	Multiple Indicators of DHS 2011
Peru	UMIC, LAC	(Hernández-Vásquez et al., 2022)	1.0 (1)	DHS 2009, 2018
Uganda	LIC, SSA	(Kakama & Basaza, 2022)	1.0 (1)	DHS 2006, 2011, 2016
Guinea	LwMIC, SSA	(Zegeye et al., 2022)	1.0 (1)	DHS 1999, 2005, 2012; MICS 2016
Cambodia	LwMIC, EAP	(Kim et al., 2022)	1.0 (1)	DHS 2005, 2010, 2014
Angola	LwMIC, SSA	(Shibre et al., 2020c)	1.0 (1)	DHS 2015
Gabon	UMIC, SSA	(Sanogo & Yaya, 2020)	1.0 (1)	DHS 2012
Egypt, Arab Rep	LwMIC, MENA	(Khadr, 2020)	1.0 (1)	DHS: 1995, 2014
**Total**	29 Countries LIC = 6 LwMIC = 16 UMIC = 7 EAP = 5 LAC = 3 MENA = 2 SA = 4 SSA = 15	104 Studies 2015 publications = 2 (1.9%) 2016 publications = 11 (10.6%) 2017 publications = 13 (12.5%) 2018 publications = 7 (6.7%) 2019 publications = 12 (11.5%) 2020 publications = 17 (16.3%) 2021 publications = 16 (15.4%) 2022 publications = 20 (19.2%) 2023 publications = 6 (5.8%)	LIC = 18.3 (19) LwMIC = 67.3 (70) UMIC = 14.4 (15)	Data year range: 1986–2021 Latest data years: 2010/2011/2012: 4 countries (LwMIC-1, LIC-1, UMIC-2) 2013/14–2015/16: 15 countries (LwMIC-10, LIC-3, UMIC-2) 2017/18–2019/2021: 10 countries (LwMIC-5, LIC-2, UMIC-3)

Panel B: Multi-country studies (n = 28)			
Countries	Income group, region	Studies reviewed	Data source
91 LMICs	Countries from all World Bank income groups and geographic regions	(Arsenault et al., 2018)	DHS: 2007–2016; MICS: 2010–2016
72 LMICs	Countries from all World Bank income groups and geographic regions	(Alkenbrack et al., 2015)	DHS: 1990–2013
72 LMICs	Countries from all World Bank income groups and geographic regions	(Boatin et al., 2018)	DHS: 2000–2004, 2010–2014; MICS: 2010–2014
72 LMICs	Countries from all World Bank income groups and geographic regions	(Lohela et al., 2019)	DHS: 1990–2016
60 LMICs	LIC (19 countries), LwMIC (31 countries) and UMIC (8 countries), HIC (2)* and World Bank geographic regions	(Wehrmeister et al., 2020)	DHS: 2010–2016; MICS: 2010–2016
48 LMICs	LIC (13 countries), LwMIC (25 countries) and UMIC (8 countries), HIC (2)* and World Bank geographic regions	(McKinnon et al., 2016)	DHS: 2006–2012
46 LMICs	LIC (12 countries), LwMIC (28 countries) and UMIC (6 countries), and World Bank geographic regions	(Wong et al., 2017)	DHS: 2003–2013
39 LMICs	LIC (17 countries), LwMIC (16 countries) and UMIC (6 countries), and World Bank geographic regions	(Anindya et al., 2021)	DHS: 2014–2018
36 LMICs	LIC (17 countries), LwMIC (13 countries) and UMIC (6 countries), and World Bank geographic regions	(Leventhal et al., 2021)	DHS: 2011–2018; MICS: 2014, 2015
30 SSA Countries	LIC (13 countries), LwMIC (14 countries) and UMIC (3 countries), and World Bank geographic regions	(Abekah-Nkrumah, 2019)	DHS: 1998–2016
28 SSA Countries	LIC (12 countries), LwMIC (14 countries) and UMIC (2 countries), and World Bank geographic regions	(Ahinkorah et al., 2022)	DHS: 2010–2020
25 SSA Countries	LIC (11 countries), LwMIC (12 countries) and UMIC (2 countries), and World Bank geographic regions	(Bobo et al., 2021a)	DHS: 2013–2018
13 Latin America and the Caribbean	LwMIC, LAC (Bolivia, Haiti, Honduras, Nicaragua) UMIC, LAC (Brazil, Guatemala, Peru, Belize, Colombia, Costa Rica, Suriname, Dominican Republic) HIC, LAC (Guyana)	(Restrepo-Méndez et al., 2015)	DHS & MICS: Bolivia (1998, 2008), Haiti (2000, 2012), Honduras (2005, 2011), Nicaragua (1997, 2001), Brazil (1996, 2006), Guatemala (1998, 2008), Peru (1996, 2012), Belize (2011), Colombia (1995, 2010), Costa Rica (2011), Suriname (2006, 2010), Dominican Republic (1996, 2007), Guyana (2006, 2009)
12 SSA Countries	LIC, SSA (Democratic Republic of Congo, Madagascar, Malawi, Mozambique) LwMIC, SSA (Angola, Eswatini, Lesotho, Tanzania, Zambia, Zimbabwe) UMIC, SSA (Namibia, South Africa)	(Selebano & Ataguba, 2021)	DHS: 2006–2016
Burundi, Ethiopia, Malawi, Rwanda, Uganda, Kenya, Tanzania, Zambia, and Zimbabwe	LIC, SSA (Burundi, Ethiopia, Malawi, Rwanda, Uganda) LwMIC, SSA (Kenya, Tanzania, Zambia, Zimbabwe)	(Bobo et al., 2021b)	DHS: Burundi (2016), Ethiopia (2016), Malawi (2016), Rwanda (2013/14), Uganda (2016), Kenya (2014), Tanzania (2015), Zambia (2018), and Zimbabwe (2015)
Ethiopia, Madagascar, Uganda, Cameroon, Zambia, and Zimbabwe	LIC, SSA (Ethiopia, Madagascar, Uganda) LwMIC, SSA (Cameroon, Zambia, Zimbabwe)	(Alam et al., 2015)	DHS: Ethiopia (2000, 2005, 2011), Madagascar (1997, 2003/04, 2008/09), Uganda (2000/01, 2006, 2011), Cameroon (1998, 2004, 2011), Zambia (1996, 2001/02, 2007), and Zimbabwe (1999, 2005/06, 2010/11)
Afghanistan, Bangladesh, India, Nepal, and Pakistan	LIC, SA (Afghanistan) LwMIC, SA (Bangladesh, India, Nepal, Pakistan)	(Rahman et al., 2017a, 2017b)	DHS: Bangladesh (2014), Afghanistan (2014); Living Condition Survey: Afghanistan (2015); HIES: Bangladesh (2010); NSSO: India (2014); District Level Household Survey: India (2012); Annual Health Survey: Nepal (2015); MICS: Nepal (2014), Pakistan (2014); Social and Living Standards Measurement Survey: Pakistan (2014)
Burkina Faso, Niger, Nigeria, Ghana, and Senegal	LIC, SSA (Burkina Faso, Niger) LwMIC, SSA (Nigeria, Ghana, Senegal)	(Ogundele et al., 2020)	DHS: Burkina Faso (2010), Niger (2012), Nigeria (2013), Ghana (2014) and Senegal (2016)
Ethiopia, Uganda, Kenya, and Tanzania	LIC, SSA (Ethiopia, Uganda) LwMIC, SSA (Kenya, Tanzania)	(Dewau et al., 2021)	DHS: Ethiopia (2016), Uganda (2016), Kenya (2014), and Tanzania (2015)
Bangladesh, Egypt, Ghana, and Zimbabwe	LwMIC, SA (Bangladesh) LwMIC, MENA (Egypt) LwMIC, SSA (Ghana, Zimbabwe)	(Hosseinpoor et al., 2016)	DHS: Bangladesh (1996, 1999, 2004, 2007), Egypt (1995, 2000, 2005, 2008), Ghana (1998, 2003, 2008), and Zimbabwe (1999, 2005, 2010)
Ethiopia, Bangladesh, Nepal, and Zimbabwe	LIC, SSA (Ethiopia) LwMIC, SSA (Zimbabwe) LwMIC, SA (Bangladesh, Nepal)	(Goli et al., 2018)	DHS: Nepal (2011), Bangladesh (2011), Ethiopia (2011), Zimbabwe (2010/11)
Ghana, Rwanda, and Philippines	LwMIC, SSA (Ghana) LwMIC, EAP (Philippines) LIC, SSA (Rwanda)	(Do et al., 2015)	DHS: Ghana (2008), Rwanda (2005), and Philippines (2008)
Bangladesh, Pakistan, and Nepal	LwMIC, SA (Bangladesh, Pakistan, Nepal)	(Huda et al., 2018)	DHS: Bangladesh (2014), Pakistan (2012/13), and Nepal (2010/11)
Brazil and Colombia	UMIC, LAC	(De La Torre et al., 2018)	DHS: Brazil (2006), and Colombia (2010)
Benin and Mali	LwMIC, SSA (Benin) LIC, SSA (Mali)	(Ravit et al., 2018)	DHS: Benin (2001, 2006, 2011/2012) and Mali (2001, 2006, 2012/13)
Ghana and Nigeria	LwMIC, SSA (Ghana and Nigeria)	(Ogundele et al., 2018)	DHS: Ghana (2003, 2008, 2014) and Nigeria (2003, 2008, 2013)
Bangladesh and Pakistan	LwMIC, SA (Bangladesh, Pakistan)	(Misu & Alam, 2023a); (Misu & Alam, 2023b)	DHS 2017/2018

LIC low-income country, LwMIC lower middle-income country, UMIC upper-middle-income country, LMIC low- and middle-income country, HIC high-income country, LAC Latin America and the Caribbean, ECA Europe and Central Asia, MENA Middle East and North Africa, SSA Sub-Saharan Africa, EAP East Asia and Pacific, SA South Asia

DHS Demographic and Health Surveys; MICS Multiple Indicator Cluster Survey; LSMS Living Standard Measurement Survey; HIES Household Income and Expenditure Survey

^a^World Bank classification of countries by income and region (https://datahelpdesk.worldbank.org/knowledgebase/articles/906519-world-bank-country-and-lending-groups)

^*^During 2023, four LwMICs (Guyana, Panama, Switzerland, and Guyana) shifted to HIC

## Study Characteristics

### Geographical Coverage

Out of the 132 studies, 78.8% (104) focused on a single country, while 21.2% (28) were multi-country studies spanning 2 to 91 LMICs (Table 1). The single-country studies encompassed 29 LMICs from all geographical regions, with 67% from LwMICs, 18% from LICs, and 14% from UMICs. India and Ethiopia hosted the majority of these studies, representing 29.8% and 2.6%, respectively. Furthermore, 6 of the 28 multi-country studies included Bangladesh, and 5 included Zimbabwe.

### Source of Data & Year of Data Collection

The studies reviewed data from 1986 to 2021, with the most recent data for 10 out of 29 countries being from 2017 or later (Table 1). Cross-sectional health survey data, such as Demographic and Health Survey (DHS), National Health Survey (NHS), and Multiple Indicator Cluster Survey (MICS) were analyzed in 97% (n = 101) of the studies.

### MHS Indicators Examined

In terms of MHS indicators (Supplementary Table 1), multiple studies have examined one, two, or more indicators of MHS (ANC, SBA, FBD, C-section delivery, and PNC). Table 2 details the distribution of studies analyzing different MHS indicators. Most studies (30.3%) assessed any three indicators, with 8.3% particularly evaluating ANC, FBD, and PNC together. ANC was the most frequently examined indicator, featured in over 68% of studies, either alone (13.6%) or with other indicators (54.6%). FBD and SBA were examined in over 40% and 41% of studies, respectively. PNC and C-section delivery were less frequently analyzed, at around 24% and 23%, respectively. Among ANC indicators (Table 3), 4 + ANC visits were the most commonly assessed, with about 41% in LICs, 48% in LwMICs, 33% in UMICs, and 53% in multi-country studies. PNC within 2 days of delivery was the most frequently accessed PNC indicator, with approximately 80% in LICs, 60% in LwMICs, and 57% in multi-country studies (Table 3).

**Table 2:** Distribution of the studies examining different Maternal Healthcare Services (MHS)

MHS Examined	Single-country studies, n (%)			Multi-country studies, n (%)	All studies, n (%)
	LIC	LwMIC	UMIC		
ANC only	1 (5.3)	10 (14.3)	4 (26.7)	3 (10.7)	18 (13.6)
FBD only	0 (0.0)	5 (7.1)	0 (0.0)	4 (14.3)	9 (6.8)
SBA only	3 (15.8)	7 (10.0)	1 (6.7)	1 (3.6)	12 (9.1)
PNC only	0 (0.0)	2 (2.9)	0 (0.0)	1 (3.6)	3 (2.3)
C-section delivery only	2 (10.5)	8 (11.4)	2 (13.3)	2 (7.1)	14 (10.6)
ANC and FBD	2 (10.5)	2 (2.9)	1 (6.7)	2 (7.1)	7 (5.3)
ANC and SBA	3 (15.8)	10 (14.3)	1 (6.7)	2 (7.1)	16 (12.1)
ANC and PNC	2 (10.5)	1 (1.4)	1 (6.7)	0 (0.0)	4 (3.0)
FBD and SBA	0 (0.0)	1 (1.4)	0 (0.0)	0 (0.0)	1 (0.8)
FBD and C-section delivery	0 (0.0)	0 (0.0)	0 (0.0)	1 (3.6)	1 (0.8)
SBA and C-section delivery	0 (0.0)	0 (0.0)	0 (0.0)	1 (3.6)	1 (0.8)
ANC, SBA and FBD	3 (15.8)	3 (4.3)	1 (6.7)	3 (10.7)	10 (7.6)
ANC, FBD and PNC	1 (5.3)	7 (10.0)	1 (6.7)	2 (7.1)	11 (8.3)
ANC, SBA, and PNC	1 (5.3)	6 (8.6)	0 (0.0)	2 (7.1)	9 (6.8)
ANC, FBD and C-section delivery	0 (0.0)	4 (5.7)	2 (13.3)	2 (7.1)	8 (6.1)
ANC, SBA, and C-section delivery	0 (0.0)	0 (0.0)	0 (0.0)	1 (3.6)	1 (0.8)
FBD, SBA, and C-section delivery	0 (0.0)	1 (1.4)	0 (0.0)	0 (0.0)	1 (0.8)
ANC, FBD, SBA, and C-section delivery	0 (0.0)	2 (2.9)	0 (0.0)	0 (0.0)	2 (1.5)
ANC, FBD, PNC, and C-section delivery	0 (0.0)	0 (0.0)	1 (6.7)	0 (0.0)	1 (0.8)
ANC, SBA, PNC, and C-section delivery	0 (0.0)	0 (0.0)	0 (0.0)	1 (3.6)	1 (0.8)
ANC, FBD, SBA and PNC	1 (5.3)	1 (1.4)	0 (0.0)	0 (0.0)	2 (1.5)
Any indicator (Total)	19 (100.0)	70 (100.0)	15 (100.0)	28 (100.0)	132 (100.0)
Any two indicators (Total)	7 (36.8)	14 (20.0)	3 (20.0)	6 (21.4)	30 (22.7)
Any three indicators (Total)	5 (26.3)	21 (30.0)	4 (26.7)	10 (35.7)	40 (30.3)
Any four indicators (Total)	1 (5.3)	3 (4.3)	1 (6.7)	1 (3.6)	6 (4.5)
ANC (Total)	14 (73.7)	46 (65.7)	12 (80.0)	18 (64.3)	90 (68.2)
FBD (Total)	7 (36.8)	26 (37.1)	6 (40.0)	14 (50.0)	53 (40.2)
SBA (Total)	11 (57.9)	31 (44.3)	3 (20.0)	10 (35.7)	55 (41.7)
PNC (Total)	5 (26.3)	17 (24.3)	3 (20.0)	6 (21.4)	31 (23.5)
C-section delivery (Total)	2 (10.5)	15 (21.4)	5 (33.3)	8 (28.6)	30 (22.7)

*ANC* Antenatal care, *PNC* Postnatal care, *FBD* Facility based delivery, *SBA* Skilled birth attendance

**Table 3:** Distribution of the studies examining ANC and PNC Indicators

MHS examined	Single-country studies, n (%)			Multi-country studies, n (%)	All studies, n (%)
	LIC	LwMIC	UMIC		
Antenatal care (ANC)					
ANC in first trimester/Early ANC	2 (9.1)	1 (1.9)	1 (5.6)	2 (7.7)	6 (5.0)
1 + ANC visit/1 + ANC visits by skilled provider	3 (13.6)	3 (5.6)	2 (11.1)	4 (15.4)	12 (10.0)
3 + ANC visits	0 (0.0)	2 (3.7)	0 (0.0)	0 (0.0)	2 (1.7)
4 + ANC visits/4 + ANC visits by skilled provider	9 (40.9)	26 (48.1)	6 (33.3)	14 (53.8)	55 (45.8)
5 + ANC visits	2 (9.1)	0 (0.0)	2 (11.1)	0 (0.0)	4 (3.3)
6 + ANC visits	0 (0.0)	0 (0.0)	1 (5.6)	0 (0.0)	1 (0.8)
7 + ANC consultations	0 (0.0)	0 (0.0)	2 (11.1)	0 (0.0)	2 (1.7)
8 + ANC contacts	0 (0.0)	1 (1.9)	0 (0.0)	1 (3.8)	2 (1.7)
ANC by skilled provider	4 (18.2)	7 (13.0)	0 (0.0)	2 (7.7)	13 (10.8)
Any ANC	1 (4.5)	4 (7.4)	2 (11.1)	0 (0.0)	7 (5.8)
Quality ANC/Full ANC	1 (4.5)	10 (18.5)	2 (11.1)	3 (11.5)	16 (13.3)
ANC (Total)	22 (100.0)	54 (100.0)	18 (100.0)	26 (100.0)	120 (100.0)
Postnatal care (PNC) of women/newborn					
1 + PNC visits/more than 1 PNC	0 (0.0)	1 (6.7)	1 (33.3)	0 (0.0)	2 (6.3)
PNC within 24 h	0 (0.0)	1 (6.7)	0 (0.0)	1 (14.3)	2 (6.3)
PNC within 2 days/48 h	4 (80.0)	9 (60.0)	0 (0.0)	4 (57.1)	17 (53.1)
PNC within 2 months	0 (0.0)	1 (6.7)	0 (0.0)	1 (14.3)	2 (6.3)
Adequate PNC	0 (0.0)	0 (0.0)	0 (0.0)	1 (14.3)	1 (3.1)
PNC within 2 weeks	0 (0.0)	1 (6.7)	0 (0.0)	0 (0.0)	1 (3.1)
PNC within 6 weeks	0 (0.0)	1 (6.7)	1 (33.3)	0 (0.0)	2 (6.3)
3 + PNC visits within 6 weeks	0 (0.0)	0 (0.0)	1 (33.3)	0 (0.0)	1 (3.1)
PNC before discharge	0 (0.0)	1 (6.7)	0 (0.0)	0 (0.0)	1 (3.1)
4 + PNC visits	1 (20.0)	0 (0.0)	0 (0.0)	0 (0.0)	1 (3.1)
Any PNC	0 (0.0)	2 (13.3)	0 (0.0)	0 (0.0)	2 (6.3)
PNC (Total)	5 (100.0)	15 (100.0)	3 (100.0)	7 (100.0)	32 (100.0)

### Measures Used to Determine Inequality in MHS Indicators

The distribution of studies determining MHS by common inequality measurements (Concentration Index, Concentration curve, Slope index of inequality, Population attributable fraction, Population attributable risk, Relative index of inequality, Between Group Variance, Coefficient of variation) is shown in Table 4. Around 81% of studies used multiple inequality measures to assess ANC inequality, with concentration index being the most frequently used measure (68%), followed by concentration curve (39%) (Table 4: Panel A). Similar trends were observed for FBD and SBA, with 81% and 80% of studies employing multiple inequality measures, respectively, and concentration index being the most commonly used measure (66% and 64%, respectively) (Table 4: Panel B-C). For C-section delivery and PNC, 90% and 84% of studies used multiple inequality measures, with concentration index being the most frequently used (63% and 68%, respectively) (Table 4: Panel D-E).

**Table 4:** Distribution of the studies by inequality measurements used for determining MHS

Inequity measurements	Single-country studies, n (%)			Multi-country studies, n (%)	All studies, n (%)
	LIC	LwMIC	UMIC		
Panel A: Inequality measurements used for determining Antenatal care (ANC)					
Concentration index (CI)	0 (0.0)	5 (10.9)	1 (8.3)	2 (11.1)	8 (8.9)
Difference	0 (0.0)	0 (0.0)	1 (8.3)	0 (0.0)	1 (1.1)
RII	0 (0.0)	1 (2.2)	0 (0.0)	0 (0.0)	1 (1.1)
Equiplot	0 (0.0)	1 (2.2)	0 (0.0)	1 (5.6)	2 (2.2)
PAF	0 (0.0)	1 (2.2)	0 (0.0)	0 (0.0)	1 (1.1)
Theil index	0 (0.0)	1 (2.2)	0 (0.0)	0 (0.0)	1 (1.1)
Blinder-Oaxaca decomposition analysis	0 (0.0)	2 (4.3)	0 (0.0)	0 (0.0)	2 (2.2)
Fairlie decomposition analysis	0 (0.0)	0 (0.0)	0 (0.0)	1 (5.6)	1 (1.1)
CC, and CI	1 (7.1)	4 (8.7)	1 (8.3)	2 (11.1)	8 (8.9)
CI, and SII	0 (0.0)	0 (0.0)	1 (8.3)	1 (5.6)	2 (2.2)
SII, and RII	0 (0.0)	0 (0.0)	0 (0.0)	2 (11.1)	2 (2.2)
Ratio, and CI	1 (7.1)	1 (2.2)	0 (0.0)	0 (0.0)	2 (2.2)
Ratio, and Difference	0 (0.0)	1 (2.2)	1 (8.3)	0 (0.0)	2 (2.2)
Ratio, and Equiplot	0 (0.0)	1 (2.2)	0 (0.0)	0 (0.0)	1 (1.1)
Theil index, and BGV	0 (0.0)	0 (0.0)	1 (8.3)	0 (0.0)	1 (1.1)
CC, and Decomposition of CI	1 (7.1)	1 (2.2)	0 (0.0)	1 (5.6)	3 (3.3)
CI, and Decomposition of CI	1 (7.1)	1 (2.2)	0 (0.0)	2 (11.1)	4 (4.4)
Equiplots, and Mean difference from the best	0 (0.0)	0 (0.0)	1 (8.3)	0 (0.0)	1 (1.1)
Theil index, and Mean difference from the best	0 (0.0)	0 (0.0)	1 (8.3)	0 (0.0)	1 (1.1)
Ratio, CC, and CI	3 (21.4)	1 (2.2)	0 (0.0)	0 (0.0)	4 (4.4)
Ratio, Difference, and CI	0 (0.0)	3 (6.5)	1 (8.3)	1 (5.6)	5 (5.6)
Ratio, Difference, and Equiplot	0 (0.0)	1 (2.2)	0 (0.0)	0 (0.0)	1 (1.1)
CI, SII, and RII	0 (0.0)	1 (2.2)	0 (0.0)	0 (0.0)	1 (1.1)
CI, SII, and Equiplots	0 (0.0)	1 (2.2)	1 (8.3)	1 (5.6)	3 (3.3)
SII, RII, and Equiplots	0 (0.0)	0 (0.0)	1 (8.3)	1 (5.6)	2 (2.2)
CC, CI, and Decomposition of CI	5 (35.7)	9 (19.6)	0 (0.0)	0 (0.0)	14 (15.6)
CC, CI, and Theil index	0 (0.0)	1 (2.2)	0 (0.0)	0 (0.0)	1 (1.1)
SII, RII, and Fairlie decomposition analysis	0 (0.0)	1 (2.2)	0 (0.0)	0 (0.0)	1 (1.1)
Ratio, Difference, PAF and PAR	0 (0.0)	3 (6.5)	0 (0.0)	0 (0.0)	3 (3.3)
Ratio, CC, CI, and multivariate decomposition analysis	1 (7.1)	0 (0.0)	0 (0.0)	0 (0.0)	1 (1.1)
CC, Gini index, Decomposition of CI, and Blinder-Oaxaca decomposition analysis	0 (0.0)	1 (2.2)	0 (0.0)	0 (0.0)	1 (1.1)
Ratio, Difference, CI, SII, and Equiplots	0 (0.0)	1 (2.2)	1 (8.3)	0 (0.0)	2 (2.2)
Ratio, Difference, CC, CI, and SII	0 (0.0)	1 (2.2)	0 (0.0)	0 (0.0)	1 (1.1)
Ratio, Difference, CI, SII, and PAR	0 (0.0)	1 (2.2)	0 (0.0)	0 (0.0)	1 (1.1)
Ratio, Difference, CI, SII, and RII	0 (0.0)	0 (0.0)	0 (0.0)	1 (5.6)	1 (1.1)
Ratio, Difference, CI, Theil index, and BGV	0 (0.0)	1 (2.2)	0 (0.0)	0 (0.0)	1 (1.1)
Ratio, Difference, CC, CI, SII, and Equiplot	1 (7.1)	0 (0.0)	0 (0.0)	1 (5.6)	2 (2.2)
Ratio, Difference, PAR, PAF, CV, Index of dissimilarity, Mean difference from mean, BGV and Theil index	0 (0.0)	0 (0.0)	0 (0.0)	1 (5.6)	1 (1.1)
Any measure (Total)	14 (100.0)	46 (100.0)	12 (100.0)	18 (100.0)	90 (100.0)
More than one measure (Total)	14 (100.0)	35 (76.1)	10 (83.3)	14 (77.8)	73 (81.1)
CI (Total)	13 (92.9)	31 (67.4)	6 (50.0)	11 (61.1)	61 (67.8)
CC (Total)	12 (85.7)	18 (39.1)	1 (8.3)	4 (22.2)	35 (38.9)
Any measure except CI (Total)	1 (7.1)	15 (32.6)	6 (50.0)	7 (38.9)	29 (32.2)

Panel B: Inequality measurements used for determining Facility-based delivery (FBD)					
Concentration index (CI)	0 (0.0)	2 (7.7)	0 (0.0)	2 (14.3)	4 (7.5)
CC	0 (0.0)	0 (0.0)	0 (0.0)	1 (7.1)	1 (1.9)
RII	0 (0.0)	1 (3.8)	0 (0.0)	0 (0.0)	1 (1.9)
Equiplot	0 (0.0)	0 (0.0)	0 (0.0)	1 (7.1)	1 (1.9)
Blinder-Oaxaca decomposition analysis	0 (0.0)	1 (3.8)	0 (0.0)	0 (0.0)	1 (1.9)
Fairlie decomposition analysis	0 (0.0)	1 (3.8)	0 (0.0)	1 (7.1)	2 (3.8)
CI, and Ratio	1 (14.3)	1 (3.8)	0 (0.0)	0 (0.0)	2 (3.8)
CC, and CI	0 (0.0)	3 (11.5)	1 (16.7)	0 (0.0)	4 (7.5)
CI, and SII	0 (0.0)	0 (0.0)	0 (0.0)	1 (7.1)	1 (1.9)
SII, and RII	0 (0.0)	0 (0.0)	0 (0.0)	1 (7.1)	1 (1.9)
CC, and Decomposition of CI	0 (0.0)	3 (11.5)	0 (0.0)	1 (7.1)	4 (7.5)
CI, and Decomposition of CI	0 (0.0)	1 (3.8)	0 (0.0)	1 (7.1)	2 (3.8)
Equiplots, and Mean difference from the best	0 (0.0)	0 (0.0)	1 (16.7)	0 (0.0)	1 (1.9)
Theil index, and BGV	0 (0.0)	0 (0.0)	1 (16.7)	0 (0.0)	1 (1.9)
Theil index, and Mean difference from the best	0 (0.0)	0 (0.0)	1 (16.7)	0 (0.0)	1 (1.9)
CC, CI, and Ratio	3 (42.9)	1 (3.8)	0 (0.0)	0 (0.0)	4 (7.5)
Ratio, Difference, and CI	0 (0.0)	2 (7.7)	1 (16.7)	1 (7.1)	4 (7.5)
CI, SII, and RII	0 (0.0)	1 (3.8)	0 (0.0)	0 (0.0)	1 (1.9)
CI, SII, and Equiplots	0 (0.0)	1 (3.8)	1 (16.7)	0 (0.0)	2 (3.8)
SII, RII, and Equiplot	0 (0.0)	0 (0.0)	0 (0.0)	1 (7.1)	1 (1.9)
CC, CI, and Decomposition of CI	3 (42.9)	3 (11.5)	0 (0.0)	0 (0.0)	6 (11.3)
CC, CI, and Theil index	0 (0.0)	1 (3.8)	0 (0.0)	0 (0.0)	1 (1.9)
SII, RII, and Fairlie decomposition analysis	0 (0.0)	1 (3.8)	0 (0.0)	0 (0.0)	1 (1.9)
CC, CI, horizontal inequity, and decomposition of CI	0 (0.0)	0 (0.0)	0 (0.0)	1 (7.1)	1 (1.9)
Oaxaca, Blinder, Reimers, and Cotton decomposition analysis	0 (0.0)	1 (3.8)	0 (0.0)	1 (7.1)	2 (3.8)
Ratio, Difference, CC, CI, and SII	0 (0.0)	1 (3.8)	0 (0.0)	0 (0.0)	1 (1.9)
Ratio, Difference, CI, Theil index, and BGV	0 (0.0)	1 (3.8)	0 (0.0)	0 (0.0)	1 (1.9)
CC, CI, Difference, Ratio, SII, and Equiplot	0 (0.0)	0 (0.0)	0 (0.0)	1 (7.1)	1 (1.9)
Any method (Total)	7 (100.0)	26 (100.0)	6 (100.0)	14 (100.0)	53 (100.0)
More than one method (Total)	7 (100.0)	21 (80.8)	6 (100.0)	9 (64.3)	43 (81.1)
CI (Total)	7 (100.0)	18 (69.2)	3 (50.0)	7 (50.0)	35 (66.0)
Any method except CI (Total)	0 (0.0)	8 (30.8)	3 (50.0)	7 (50.0)	18 (34.0)

Panel C: Inequality measurements used for determining Skilled birth attendance (SBA)					
CI	0 (0.0)	2 (6.5)	0 (0.0)	1 (10.0)	3 (5.5)
Gini coefficient	0 (0.0)	1 (3.2)	0 (0.0)	0 (0.0)	1 (1.8)
SII	0 (0.0)	0 (0.0)	1 (33.3)	0 (0.0)	1 (1.8)
Equiplot	0 (0.0)	1 (3.2)	0 (0.0)	1 (10.0)	2 (3.6)
PAF	0 (0.0)	1 (3.2)	0 (0.0)	0 (0.0)	1 (1.8)
Theil index	0 (0.0)	1 (3.2)	0 (0.0)	0 (0.0)	1 (1.8)
Oaxaca decomposition analysis	0 (0.0)	1 (3.2)	0 (0.0)	0 (0.0)	1 (1.8)
Multivariate decomposition analysis	0 (0.0)	1 (3.2)	0 (0.0)	0 (0.0)	1 (1.8)
CI, and Ratio	3 (27.3)	0 (0.0)	0 (0.0)	0 (0.0)	3 (5.5)
CC, and CI	0 (0.0)	1 (3.2)	0 (0.0)	1 (10.0)	2 (3.6)
CI, and Equiplot	0 (0.0)	0 (0.0)	0 (0.0)	1 (10.0)	1 (1.8)
Ratio, and Difference	0 (0.0)	1 (3.2)	0 (0.0)	0 (0.0)	1 (1.8)
CC, and Decomposition of CI	0 (0.0)	1 (3.2)	0 (0.0)	0 (0.0)	1 (1.8)
CI, and Decomposition of CI	1 (9.1)	1 (3.2)	0 (0.0)	1 (10.0)	3 (5.5)
Equiplots, and Mean difference from the best	0 (0.0)	0 (0.0)	1 (33.3)	0 (0.0)	1 (1.8)
Horizontal inequity, and Decomposition of CI	1 (9.1)	0 (0.0)	0 (0.0)	0 (0.0)	1 (1.8)
CC, CI, and Ratio	1 (9.1)	1 (3.2)	0 (0.0)	0 (0.0)	2 (3.6)
CI, SII, and Equiplot	0 (0.0)	0 (0.0)	0 (0.0)	1 (10.0)	1 (1.8)
CI, SII, and RII	0 (0.0)	1 (3.2)	0 (0.0)	0 (0.0)	1 (1.8)
SII, RII, and Equiplots	0 (0.0)	0 (0.0)	1 (33.3)	1 (10.0)	2 (3.6)
Difference, CC, and CI	1 (9.1)	0 (0.0)	0 (0.0)	0 (0.0)	1 (1.8)
Ratio, Difference, and Equiplot	0 (0.0)	1 (3.2)	0 (0.0)	0 (0.0)	1 (1.8)
CC, CI, and Decomposition of CI	3 (27.3)	8 (25.8)	0 (0.0)	0 (0.0)	11 (20.0)
Ratio, Difference, PAF, and PAR	0 (0.0)	3 (9.7)	0 (0.0)	0 (0.0)	3 (5.5)
CC, Gini index, Decomposition of CI, and Oaxaca-blinder decomposition analysis	0 (0.0)	1 (3.2)	0 (0.0)	0 (0.0)	1 (1.8)
Ratio, Difference, CC, CI, and SII	0 (0.0)	1 (3.2)	0 (0.0)	0 (0.0)	1 (1.8)
Ratio, Difference, CI, SII, and Equiplots	1 (9.1)	1 (3.2)	0 (0.0)	1 (10.0)	3 (5.5)
Ratio, Difference, CI, SII, and PAR	0 (0.0)	1 (3.2)	0 (0.0)	0 (0.0)	1 (1.8)
Ratio, Difference, CI, Theil index, and BGV	0 (0.0)	1 (3.2)	0 (0.0)	0 (0.0)	1 (1.8)
CC, CI, Difference, Ratio, SII, and Equiplot	0 (0.0)	0 (0.0)	0 (0.0)	1 (10.0)	1 (1.8)
Ratio, Difference, PAR, PAF, Coefficient of variation, Index of dissimilarity, Mean difference from mean, BGV and Theil index	0 (0.0)	0 (0.0)	0 (0.0)	1 (10.0)	1 (1.8)
Any method (Total)	11 (100.0)	31 (100.0)	3 (100.0)	10 (100.0)	55 (100.0)
More than one method (Total)	11 (100.0)	23 (74.2)	2 (66.7)	8 (80.0)	44 (80.0)
CI (Total)	10 (90.0)	18 (58.1)	0 (0.0)	7 (70.0)	35 (63.6)
Any method except CI (Total)	1 (9.1)	13 (41.9)	3 (100.0)	3 (30.0)	20 (36.4)

Panel D: Inequality measurements used for determining C-section delivery					
CC	0 (0.0)	0 (0.0)	0 (0.0)	1 (12.5)	1 (3.3)
Fairlie decomposition analysis	0 (0.0)	0 (0.0)	0 (0.0)	1 (12.5)	1 (3.3)
Multivariate decomposition analysis	0 (0.0)	0 (0.0)	0 (0.0)	1 (12.5)	1 (3.3)
CC, and CI	0 (0.0)	4 (26.7)	0 (0.0)	1 (12.5)	5 (16.7)
SII, and RII	0 (0.0)	0 (0.0)	0 (0.0)	1 (12.5)	1 (3.3)
CC, and Gini coefficient	0 (0.0)	1 (6.7)	0 (0.0)	0 (0.0)	1 (3.3)
CI, and Equiplot	0 (0.0)	0 (0.0)	0 (0.0)	1 (12.5)	1 (3.3)
Ratio, and Difference	0 (0.0)	0 (0.0)	1 (20.0)	0 (0.0)	1 (3.3)
CI, and Decomposition of CI	0 (0.0)	1 (6.7)	0 (0.0)	1 (12.5)	2 (6.7)
Theil index, and BGV	0 (0.0)	0 (0.0)	1 (20.0)	0 (0.0)	1 (3.3)
CC, CI, and Ratio	0 (0.0)	1 (6.7)	0 (0.0)	0 (0.0)	1 (3.3)
Ratio, Difference, and CI	0 (0.0)	2 (13.3)	1 (20.0)	0 (0.0)	3 (10.0)
CI, SII, and Equiplot	0 (0.0)	0 (0.0)	1 (20.0)	0 (0.0)	1 (3.3)
CC, CI, and Decomposition of CI	0 (0.0)	4 (26.7)	0 (0.0)	0 (0.0)	4 (13.3)
Difference, PAR and SII	1 (50.0)	0 (0.0)	0 (0.0)	0 (0.0)	1 (3.3)
Ratio, PAR, SII, and CI	1 (50.0)	0 (0.0)	0 (0.0)	0 (0.0)	1 (3.3)
Ratio, Difference, Gini index, and Equiplot	0 (0.0)	0 (0.0)	0 (0.0)	1 (12.5)	1 (3.3)
Ratio, Difference, SII, and RII	0 (0.0)	1 (6.7)	0 (0.0)	0 (0.0)	1 (3.3)
Ratio, Difference, PAF, and PAR	0 (0.0)	1 (6.7)	0 (0.0)	0 (0.0)	1 (3.3)
CI, SII, RII and Equiplot	0 (0.0)	0 (0.0)	1 (20.0)	0 (0.0)	1 (3.3)
Any method (Total)	2 (100.0)	15 (100.0)	5 (100.0)	8 (100.0)	30 (100.0)
More than one method (Total)	2 (100.0)	15 (100.0)	5 (100.0)	5 (62.5)	27 (90.0)
CI (Total)	1 (50.0)	12 (80.0)	3 (60.0)	3 (37.5)	19 (63.3)
Any method except CI (Total)	1 (50.0)	3 (20.0)	2 (40.0)	5 (62.5)	11 (36.7)

Panel E: Inequality measurements used for determining Postnatal care of women/newborn					
CI	0 (0.0)	2 (11.8)	1 (33.3)	0 (0.0)	3 (9.7)
PAR	0 (0.0)	1 (5.9)	0 (0.0)	0 (0.0)	1 (3.2)
Blinder-Oaxaca decomposition analysis	0 (0.0)	1 (5.9)	0 (0.0)	0 (0.0)	1 (3.2)
CC, and CI	1 (20.0)	2 (11.8)	1 (33.3)	0 (0.0)	4 (12.9)
CI, and SII	0 (0.0)	0 (0.0)	0 (0.0)	1 (16.7)	1 (3.2)
Ratio, and Equiplot	0 (0.0)	1 (5.9)	0 (0.0)	0 (0.0)	1 (3.2)
Theil index, and BGV	0 (0.0)	0 (0.0)	1 (33.3)	0 (0.0)	1 (3.2)
CC, and Decomposition of CI	1 (20.0)	2 (11.8)	0 (0.0)	1 (16.7)	4 (12.9)
CI, and Decomposition of CI	0 (0.0)	0 (0.0)	0 (0.0)	1 (16.7)	1 (3.2)
CC, CI, and Ratio	2 (40.0)	0 (0.0)	0 (0.0)	0 (0.0)	2 (6.5)
CI, SII, and Equiplot	0 (0.0)	1 (5.9)	0 (0.0)	1 (16.7)	2 (6.5)
CC, CI, and Decomposition of CI	1 (20.0)	3 (17.6)	0 (0.0)	0 (0.0)	4 (12.9)
CC, CI, and Theil index	0 (0.0)	1 (5.9)	0 (0.0)	0 (0.0)	1 (3.2)
SII, RII, and Fairlie decomposition analysis	0 (0.0)	1 (5.9)	0 (0.0)	0 (0.0)	1 (3.2)
CC, Gini index, Decomposition of CI, and Blinder-Oaxaca decomposition analysis	0 (0.0)	1 (5.9)	0 (0.0)	0 (0.0)	1 (3.2)
Ratio, Difference, CC, CI, and SII	0 (0.0)	1 (5.9)	0 (0.0)	0 (0.0)	1 (3.2)
Ratio, Difference, CI, SII, and RII	0 (0.0)	0 (0.0)	0 (0.0)	1 (16.7)	1 (3.2)
Ratio, Difference, CC, CI, SII, and Equiplot	0 (0.0)	0 (0.0)	0 (0.0)	1 (16.7)	1 (3.2)
Any method (Total)	5 (100.0)	17 (100.0)	3 (100.0)	6 (100.0)	31 (100.0)
More than one method (Total)	5 (100.0)	13 (76.5)	2 (66.7)	6 (100.0)	26 (83.9)
CI (Total)	4 (80.0)	10 (58.8)	2 (66.7)	5 (83.3)	21 (67.7)
Any method except CI (Total)	1 (20.0)	7 (41.2)	1 (33.3)	1 (16.7)	10 (32.3)

*CI* Concentration Index, *CC* Concentration curve, *SII* Slope index of inequality, *PAF* Population attributable fraction, *PAR* Population attributable risk, *RII* Relative index of inequality, *BGV* Between Group Variance, *CV* Coefficient of variation

### Equity Strata Used to Assess Inequality in MHS Indicators

Table 5 presents how frequently different common equity strata (e.g., wealth, education, residence) were used across studies evaluating inequality in various MHS indicators among single-country studies. Wealth status was the most commonly assessed equity stratum, featured in 97 studies using any inequality measure and 62 studies using the concentration index. This was followed by area of residence (64 and 36 studies, respectively) and women’s education (61 and 32 studies). Antenatal care (ANC) was the most frequently studied MHS, with 66 studies assessing wealth-related inequality and 50 using the concentration index. Other equity dimensions were less frequently explored, with region appearing in 40 studies, women’s age in 36, religion in 16, and ethnicity in only 7 studies.

**Table 5:** Distribution of studies in common equity strata by maternal health service (MHS) indicators

Equity strata	Number of studies for different MHS indicators					
	ANC	FBD	SBA	C-section delivery	PNC	Any MHS indicators
Panel A: Distribution of studies in common equity strata by MHS indicators using any inequality measure in single-country studies						
Wealth status	66	37	41	21	24	97
Area of Residence	40	23	29	15	14	64
Women’s education	35	18	28	11	12	61
Region	20	13	15	13	6	40
Women’s age	23	15	15	5	10	36
Religion	10	5	9	3	5	16
Ethnicity	4	2	4	0	1	7
Panel B: Distribution of studies in common equity strata by MHS indicators using concentration index in single-country studies						
Wealth status	50	31	28	16	19	62
Area of Residence	28	19	19	10	9	36
Women’s education	24	13	19	7	7	32
Region	14	9	11	8	4	17
Women’s age	16	8	13	5	5	20
Religion	6	3	4	3	2	9

### Evidence of Wealth-Related Inequality in MHS in LMICs

Table 6 shows wealth-related inequality in five MHS indicators by concentration index for single-country studies. In Ethiopia, the concentration index of 4 + ANC visits, with varying sample sizes, was 0.41 in 2000 and 2005, dropped to 0.38 in 2014, and remained at 0.26 in 2016. In Nepal, the concentration index for 4 + ANC visits was 0.60 in 1996, declined from 2001 to 2011, and reached 0.08 in 2016. In Brazil, the concentration index for 4 + ANC visits was 0.11 in 1996 and nearly zero (0.02) in 2013. In Bangladesh, the concentration index of FBD was 0.65 in 1994, fell to 0.41 in 2011, and rose slightly to 0.47 in 2014. In Nigeria, the concentration index of FBD ranged from 0.59 to 0.69 between 2003 and 2017, gradually decreasing in the latest year. In Indonesia, the concentration index for FBD ranged from 0.53 in 1989 to 0.15 in 2012. In Sierra Leone, the concentration index for SBA was 0.33 in 2008 and declined to 0.11 in 2019. In India, the concentration index for SBA ranged from 0.27 to 0.55 in 2006 and from 0.08 to 0.24 in 2016. In Ghana, the concentration index for SBA was 0.60 in 2003, declined in subsequent years, and ranged from 0.19 to 0.42 in 2014.

**Table 6:** Wealth-related inequality in MHS indicators by concentration index (CI) in single-country studies

Country	Year	4 + ANC visits		FBD		SBA		C-section	
		CI	Sample size	CI	Sample size	CI	Sample size	CI	Sample size
Lower-income counties									
Afghanistan	2010			0.31	15,688	0.31	21,290		
	2011	0.36	21,290						
Ethiopia	2000	0.41	7917	0.49	7917	0.48—0.67	7917—15,000	0.78	15,367
	2005	0.41	7273	0.48	7273	0.52—0.65	7273	0.70	14,070
	2011	0.35	7500	0.50	7500	0.53—0.59	7836	0.68	16,515
	2014	0.38	5710	0.35	5710	0.53	7500—8070		
	2016					0.30—0.57	10,641—16,515	0.47	15,683
Sierra Leone	2008	0.20	3380	0.20	5651	0.33	5811		
	2013	0.05	7532	0.20	12,079	0.25	12,198		
	2019	0.02	6448	0.11	9771	0.11	9771		
Uganda	2006			0.20	8536	0.19	8531		
	2011			0.12	8674	0.12	8674		
	2016			− 0.72	18,506	− 0.72	18,506		
Lower middle-income counties									
Bangladesh	1994			0.65	7608				
	1998			0.56	7589				
	2002			0.49	7407				
	2004	0.42	3730	0.49	3730	0.47	3730	0.58–0.60	3730–5359
	2006			0.48	7073				
	2007	0.32	3365	0.41	3365	0.42	3365	0.48	3365
	2011	0.23–0.031	1323–4648	0.33–0.41	4638–8759	0.34–0.44	4638–4648	0.31–0.40	4638–4648
	2014	0.25–0.33	1435–4483	0.47	4481–4483	0.47	4481–4483	0.30–0.38	4481–4486
Egypt	1995	0.51	7532						
	2014	0.17	10,864						
Ghana	2003	0.30	5691			0.60	5691		
	2008	0.26	4916			0.56	4916		
	2011			0.29	10,963				
	2014	0.18	9396			0.19–0.42	1305–9396	0.17	4294
India	1993							0.49	
	1999							0.47	
	2006	0.29–0.33	36,850–109,041	0.31–0.33	36,850–109,041	0.27–0.55	34,560–36,850	0.48–0.49	51,555
	2016	0.19–0.20	186,721–601,509	0.07–0.10	87,975–601,509	0.08–0.24	178,857–186,721	0.36	249,949
	2021							0.29	232,920
Kenya	2014	0.11	14,741			0.22	14,741		
Nepal	1996	0.60	4417	0.65	4,417			0.64	4417
	2001	0.44–0.58	6931	0.66	6,931	0.47		0.76	6931
	2006	0.31–0.42	5783	0.57	5,783	0.44		0.77	5783
	2011	0.21–0.31	5306	0.40	5,306	0.33		0.66	5306
	2016	0.08				0.15			
Nigeria	1990					0.31	8999		
	2003	0.60	4933	0.69	4,933	0.51	4933		
	2008	0.72	6561	0.67	6,561	0.43–0.57	6561–33,385		
	2013	0.54–0.58	6756–18,559	0.60	6,756	0.49	6756	0.49	20,468
	2017	0.51	6704	0.59	6,704	0.66	6704		
Mauritania	2007					0.63	3539		
	2015					0.59	4172		
Philippines	2008	0.38						0.55	
	2013	0.21						0.52	
Tanzania	2004	0.18	5593	0.20	8725	0.20	8725		
	2010	0.18	5404	0.21	8176	0.21	8176		
	2016	0.24	6937	0.18	10,052	0.17	10,052		
Vietnam	2014	0.19	9979	0.06–0.07	1473–9979	0.06	9979		
Zimbabwe	2015	0.09	4595			0.05	4595		
Upper middle-income counties									
Brazil	1986			0.13				0.36	
	1996	0.11		0.05				0.28	
	2006	0.03		0.01				0.19	
	2013	0.02		0.01				0.17	
Gabon	2012	0.12	8422	0.07	8422				
Indonesia	1989	0.20	11,929	0.53	11,929				
	1993	0.16	23,517	0.47	23,517				
	1997	0.12	15,983	0.40	15,983				
	2001	0.10	12,837	0.35	12,837				
	2005	0.10	15,684	0.35	15,684				
	2009	0.08	15,226	0.21	15,226				
	2012	0.06	9044	0.15	9044				

*ANC* Antenatal care, *FBD* Facility-based delivery, *SBA* Skilled birth attendance, *CI* Concentration index

## Discussion

This scoping review compiles recent empirical evidence on inequality in MHS in LMICs. Compared to prior pertinent reviews by (Langlois et al., 2015) and (Jongh et al., 2016), the number of studies in LMICs has grown substantially since 2015. The adoption of the SDGs by the United Nations in 2015, with a particular focus on improving health and well-being (SDG 3), dropping maternal mortality (SDG 3.1), and reducing inequality within and between countries (SDG 10), most likely boosted the literature growth.

Nevertheless, our review identified substantial gaps in the research. The country coverage of the LMIC literature was skewed towards India and Ethiopia. While not directly comparable, an earlier review of developing countries noted that literature coverage was predominantly concentrated in India and Bangladesh until 2015 (Çalışkan et al., 2015). Few studies (one or two) addressed inequality in MHS indicators in LICs like Afghanistan, Sierra Leone, Burundi, Togo, and Uganda. No studies assessed all five common MHS indicators together. Only one study in LIC (Ethiopia), three in LwMIC (India and Bangladesh), and one in UMIC (China) examined four indicators collectively. PNC and C-section delivery were less frequently analyzed compared to ANC, FBD, and SBA. Similarly, a review study in developing countries up to 2015 found PNC less frequently examined than ANC, delivery care, and SBA (Çalışkan et al., 2015). ANC provides effective interventions to mitigate pregnancy and childbirth risks and serves as a platform for delivering additional health services (Lawn & Kerber, 2016). Skilled birth attendance is crucial for saving early neonatal lives and typically necessitates care in a health facility (Bhutta et al., 2014; Campbell et al., 2016). These might instigate the growth of more studies on ANC, FBA and SBA services.

Most of the studies were cross-sectional in nature and analyzed the DHS. To assess inequality in MHS indicators, most studies used multiple inequality measures recommended by the World Bank or WHO, with concentration index being the most frequently applied. The frequent use of the concentration index approach can be attributed to its ability to provide a nuanced understanding of inequality by considering disparities across the entire population, which is particularly relevant in maternal health where inequalities often exist across multiple socioeconomic gradients (Bintabara & Mwampagatwa, 2023; O'Donnell et al., 2008).

Our findings underscore a predominant focus on economic (wealth) and geographic (residence, region) inequalities, while sociocultural factors (e.g., religion, ethnicity) remain underexplored. This imbalance may stem from the fact that economic and geographic disparities are more directly linked to structural barriers such as affordability, service availability, and distance to facilities, making them easier to measure and more actionable for policy (La Barbera, 2023; Majebi et al., 2024; Morgan & M Breau, 2024). In contrast, religion and ethnicity are often less consistently collected and politically sensitive, limiting their use in large-scale studies (Kachoria et al., 2022; Krieger, 2012). Wealth-related inequality emerged as the most commonly assessed equity stratum, with evidence consistently showing that women in the poorest wealth quintiles were significantly less likely to utilize any MHS. Though not directly comparable due to methodological differences, a recent review identified education as a frequent barrier to MHS utilization in low- and lower-middle-income countries (LLMICs) (Sarikhani et al., ﻿^2024^). Differences in findings may result from the specific focus on LLMICs and varying analysis periods.

Despite substantial improvements in reducing inequality between the poorest and richest women across five MHS indicators in almost all LMICs, inequalities still persist. Wealth-related disparities were most prominent in Nigeria for 4 + ANC visits, facility-based delivery, skilled birth attendance, and C-section deliveries, followed by Ethiopia, Bangladesh, Nepal, Ghana, Mauritania, India, and the Philippines, among all LMICs in the latest study years. These findings illustrate that inequality in MHS indicators was most severe in LICs and LwMICs. A recent study indicates Nigeria had the highest levels of wealth-related inequality in ANC, FBD, and PNC services among 17 sub-Saharan African countries (Asefa et al., 2023). Policymakers and health administrators should adopt successful country-specific interventions to reduce MHS inequalities in the most impoverished countries (Leventhal et al., 2021).

In light of the gaps observed in the literature, we suggest future research should prioritize PNC and C-section deliveries, as these areas have been underexplored in LMICs. The postpartum period is critical since, in LMICs, the risk of women dying during the postpartum period is significantly higher (WHO, 2019). Medically indicated C-section is a life-saving intervention for mothers and infants (Boerma et al., 2018). Utilizing both cross-sectional and panel data will provide a clearer picture of inequality in MHS over time (Zhao et al., 2020). A comprehensive investigation of all five MHS indicators will reveal the overall unequal utilization of services across different population groups, each offering crucial, life-saving benefits for preventing childbirth-related complications (Kitila et al., 2022). Policymakers should adopt equity-oriented health policies to ensure the well-being of pregnant women from early pregnancy through the postpartum phase. Employing various inequality measures alongside the concentration index is essential to check the robustness of the study results. Future research must focus on LICs, particularly where recent studies on MHS inequality are lacking. Although global studies cover many LICs, they rarely address country-specific inequality issues and their drivers. Understanding these drivers at the country level would enable targeted resource allocation in MHS to meet SDG targets by reducing inequality among population groups (Ward et al., 2023).

Our study’s strength lies in examining all inequality studies of MHS in LMICs from the imposition of SDGs in 2015 to the present. Additionally, we include studies that utilize both simple and complex inequality measures recommended by the World Bank or WHO. Our study is more comprehensive than previous ones, which have not considered all five common MHS indicators in LMICs. Despite these strengths, there are limitations to note. The key limitation of this review is that we omitted grey literature, or studies published in a language other than English. Hence, we may have overlooked some potentially relevant national studies which could have expanded the knowledge base. Additionally, the review primarily assessed inequality level concerning the most commonly used equity stratum (wealth), potentially limiting insights into inequalities involving less frequently analyzed yet important equity strata.

## Conclusion

In this study, we critically reviewed 132 studies on the five common MHS indicators in LMICs, published between 2015 and mid-2023. Despite the incredible growth in literature since 2015, notable gaps remain in MHS health inequality research. Firstly, there are limited studies on LICs and UMICs, even though each LMIC region has at least some research on any of the MHS indicators. Additionally, almost all the studies used cross-sectional studies, and a large volume of studies are focused on India and Ethiopia. Secondly, no such study has assessed all the five common indicators of MHS altogether. While ANC is the most examined indicator, the inequality assessment is less frequent in PNC and C-section delivery. Thirdly, among all the simple and complex inequality measures, concentration index is the most frequently used inequality measure. Most studies prioritize the assessment of economic and geographic disparities over sociocultural inequalities. Future research on MHS should thoroughly address the gaps in utilization among population groups, considering all the common MHS indicators, especially in LICs, applying both simple and complex inequality measures, and exploring a broader range of equity strata. More research is required to analyze inequality in the PNC and C-section delivery in LICs.

## Supplementary Information

Below is the link to the electronic supplementary material.

Supplementary file1 (DOCX 19 KB)

Supplementary file2 (DOCX 51 KB)

## Data Availability

This article and the supplementary files contain all the data that were analyzed for this study.

## References

[CR1] Abekah-Nkrumah, G. (2018). Spatial variation in the use of reproductive health services over time: A decomposition analysis. *BMC Pregnancy and Childbirth*. 10.1186/s12884-018-1695-3 29510675 10.1186/s12884-018-1695-3 PMC5838884

[CR2] Abekah-Nkrumah, G. (2019). Trends in utilisation and inequality in the use of reproductive health services in Sub-Saharan Africa. *BMC Public Health*. 10.1186/s12889-019-7865-z 31752773 10.1186/s12889-019-7865-z PMC6873654

[CR3] Adeyanju, O., Tubeuf, S., & Ensor, T. (2017). Socio-economic inequalities in access to maternal and child healthcare in Nigeria: Changes over time and decomposition analysis. *Health Policy and Planning*. 10.1093/heapol/czx049 28520949 10.1093/heapol/czx049

[CR4] Adusumilli, G., Pederson, J. M., Hardy, N., Kallmes, K. M., Hutchison, K., Kobeissi, H., Heiferman, D. M., Kallmes, D., Brinjikji, W., Albers, G. W., & Heit, J. J. (2022). Endovascular therapy versus medical therapy alone for basilar artery stroke: A systematic review and meta-analysis through nested knowledge. *Stroke: Vascular and Interventional Neurology*. 10.1161/SVIN.121.000147 10.1161/SVIN.121.000147 PMC12778767 41583624

[CR5] Agho, K. E., Ezeh, O. K., Issaka, A. I., Enoma, A. I., Baines, S., & Renzaho, A. M. N. (2016). Population attributable risk estimates for factors associated with non-use of postnatal care services among women in Nigeria. *British Medical Journal Open*. 10.1136/bmjopen-2015-010493 10.1136/bmjopen-2015-010493 PMC4947753 27371552

[CR6] Ahinkorah, B. O., Aboagye, R. G., Seidu, A.-A., Okyere, J., Mohammed, A., Chattu, V. K., Budu, E., Adoboi, F., & Yaya, S. (2022). Rural-urban disparities in caesarean deliveries in sub-Saharan Africa: A multivariate non-linear decomposition modelling of demographic and health survey data. *BMC Pregnancy and Childbirth*. 10.1186/s12884-022-04992-6 36115842 10.1186/s12884-022-04992-6 PMC9482294

[CR7] Akseer, N., Bhatti, Z., Rizvi, A., Salehi, A. S., Mashal, T., & Bhutta, Z. A. (2016). Coverage and inequalities in maternal and child health interventions in Afghanistan. *BMC Public Health*. 10.1186/s12889-016-3406-1 27634540 10.1186/s12889-016-3406-1 PMC5025831

[CR8] Alam, N., Hajizadeh, M., Dumont, A., & Fournier, P. (2015). Inequalities in maternal health care utilization in sub-Saharan African countries: A multiyear and multi-country analysis. *PLoS ONE*. 10.1371/journal.pone.0120922 25853423 10.1371/journal.pone.0120922 PMC4390337

[CR9] Ali, B., Debnath, P., & Anwar, T. (2021). Inequalities in utilisation of maternal health services in urban India: Evidences from national family health survey-4. *Clinical Epidemiology and Global Health*. 10.1016/j.cegh.2020.11.00534179566

[CR10] Ali, B., Dhillon, P., & Mohanty, S. K. (2020). Inequalities in the utilization of maternal health care in the pre- and post-National Health Mission periods in India. *Journal of Biosocial Science*. 10.1017/s0021932019000385 31232249 10.1017/S0021932019000385

[CR11] Ali, S., Thind, A., Stranges, S., Campbell, M. K., & Sharma, I. (2023). Investigating health inequality using trend, decomposition and spatial analyses: A study of maternal health service use in Nepal. *International Journal of Public Health*. 10.3389/ijph.2023.1605457 37332772 10.3389/ijph.2023.1605457 PMC10272384

[CR12] Alkenbrack, S., Chaitkin, M., Zeng, W., Couture, T., & Sharma, S. (2015). Did equity of reproductive and maternal health service coverage increase during the MDG Era? An analysis of trends and determinants across 74 Low- and middle-income countries. *PLoS ONE*. 10.1371/journal.pone.0134905 26331846 10.1371/journal.pone.0134905 PMC4558013

[CR13] Ambel, A. A., Andrews, C., Bakilana, A. M., Foster, E. M., Khan, Q., & Wang, H. (2017). Examining changes in maternal and child health inequalities in Ethiopia. *International Journal for Equity in Health*. 10.1186/s12939-017-0648-1 28830454 10.1186/s12939-017-0648-1 PMC5568328

[CR14] Amporfu, E., & Grépin, K. A. (2019). Measuring and explaining changing patterns of inequality in institutional deliveries between urban and rural women in Ghana: A decomposition analysis. *International Journal for Equity in Health*. 10.1186/s12939-019-1025-z 31399050 10.1186/s12939-019-1025-z PMC6688245

[CR15] Anarwat, S. G., Salifu, M., & Akuriba, M. A. (2021). Equity and access to maternal and child health services in Ghana a cross-sectional study. *BMC Health Services Research*. 10.1186/s12913-021-06872-9 34425805 10.1186/s12913-021-06872-9 PMC8383376

[CR16] Anindya, K., Marthias, T., Vellakkal, S., Carvalho, N., Atun, R., Morgan, A., Zhao, Y., Hulse, E. S., McPake, B., & Lee, J. T. (2021). Socioeconomic inequalities in effective service coverage for reproductive, maternal, newborn, and child health: A comparative analysis of 39 low-income and middle-income countries. *EClinicalMedicine*. 10.1016/j.eclinm.2021.101103 34527893 10.1016/j.eclinm.2021.101103 PMC8430373

[CR17] Anwar, I., Nababan, H. Y., Mostari, S., Rahman, A., & Khan, J. A. M. (2015). Trends and inequities in use of maternal health care services in Bangladesh, 1991–2011. *PLoS ONE*. 10.1371/journal.pone.0120309 25799500 10.1371/journal.pone.0120309 PMC4370698

[CR18] Arsenault, C., Jordan, K., Lee, D., Dinsa, G., Manzi, F., Marchant, T., & Kruk, M. E. (2018). Equity in antenatal care quality: An analysis of 91 national household surveys. *The Lancet Global Health*. 10.1016/s2214-109x(18)30389-9 30322649 10.1016/S2214-109X(18)30389-9 PMC6187112

[CR19] Asamoah, B. O., & Agardh, A. (2017). Inequality trends in maternal health services for young Ghanaian women with childbirth history between 2003 and 2014. *British Medical Journal Open*. 10.1136/bmjopen-2016-011663 10.1136/bmjopen-2016-011663 PMC5306510 28174219

[CR20] Asefa, A., Gebremedhin, S., Marthias, T., Nababan, H., Christou, A., Semaan, A., Banke-Thomas, A., Tabana, H., Al-beity, F. M. A., Dossou, J.-P., Gutema, K., Delvaux, T., Birabwa, C., Dennis, M., Grovogui, F. M., McPake, B., & BeÅová, L. (2023). Wealth-based inequality in the continuum of maternal health service utilisation in 16 sub-Saharan African countries. *International Journal for Equity in Health,**22*(1), 1–203. 10.1186/s12939-023-02015-0 37784140 10.1186/s12939-023-02015-0 PMC10544383

[CR21] Atake, E.-H. (2021). Socio-economic inequality in maternal health care utilization in Sub-Saharan Africa: Evidence from Togo. *International Journal of Health Planning and Management*. 10.1002/hpm.3083 33000498 10.1002/hpm.3083

[CR22] Banke-Thomas, O. E., Banke-Thomas, A. O., & Ameh, C. A. (2017). Factors influencing utilisation of maternal health services by adolescent mothers in Low-and middle-income countries: A systematic review. *BMC Pregnancy and Childbirth,**17*(1), 65–65. 10.1186/s12884-017-1246-3 28209120 10.1186/s12884-017-1246-3 PMC5314631

[CR23] Bayati, M., Feyzabadi, V. Y., & Rashidian, A. (2017). Geographical disparities in the health of Iranian women: Health outcomes, behaviors, and health-care access indicators. *International Journal of Preventive Medicine*. 10.4103/ijpvm.ijpvm_67_16 28348721 10.4103/ijpvm.IJPVM_67_16 PMC5353772

[CR24] Bhusal, U. P. (2021). Predictors of wealth-related inequality in institutional delivery: A decomposition analysis using Nepal multiple Indicator cluster survey (MICS) 2019. *BMC Public Health*. 10.1186/s12889-021-12287-2 34893047 10.1186/s12889-021-12287-2 PMC8665495

[CR25] Bhutta, Z. A. P., Das, J. K. M. B. A., Bahl, R. P., Lawn, J. E. P., Salam, R. A. M., Paul, V. K. M. D., Sankar, M. J. D. M., Blencowe, H. P., Rizvi, A. M., Chou, V. B. P., & Walker, N. P. (2014). Can available interventions end preventable deaths in mothers, newborn babies, and stillbirths, and at what cost? *The Lancet (British Edition),**384*(9940), 347–370. 10.1016/S0140-6736(14)60792-3 10.1016/S0140-6736(14)60792-3 24853604

[CR26] Bintabara, D. (2021). Addressing the huge poor-rich gap of inequalities in accessing safe childbirth care: A first step to achieving universal maternal health coverage in Tanzania. *PLoS ONE*. 10.1371/journal.pone.0246995 33592017 10.1371/journal.pone.0246995 PMC7886203

[CR27] Bintabara, D., & Basinda, N. (2021). Twelve-year persistence of inequalities in antenatal care utilisation among women in Tanzania: A decomposition analysis of population-based cross-sectional surveys. *British Medical Journal Open*. 10.1136/bmjopen-2020-040450 10.1136/bmjopen-2020-040450 PMC8061846 33879479

[CR28] Bintabara, D., & Mwampagatwa, I. (2023). Socioeconomic inequalities in maternal healthcare utilization: An analysis of the interaction between wealth status and education, a population-based surveys in Tanzania. *PLOS Global Public Health,**3*(6), e0002006–e0002006. 10.1371/journal.pgph.0002006 37310944 10.1371/journal.pgph.0002006 PMC10263315

[CR29] Boatin, A. A., Schlotheuber, A., Betran, A. P., Moller, A.-B., Barros, A. J. D., Boerma, T., Torloni, M. R., Victora, C. G., & Hosseinpoor, A. R. (2018). Within country inequalities in caesarean section rates: Observational study of 72 low and middle income countries. *BMJ*. 10.1136/bmj.k55 29367432 10.1136/bmj.k55 PMC5782376

[CR30] Bobo, F. T., Asante, A., Woldie, M., Dawson, A., & Hayen, A. (2021a). Spatial patterns and inequalities in skilled birth attendance and caesarean delivery in sub-Saharan Africa. *BMJ Global Health*. 10.1136/bmjgh-2021-007074 34716145 10.1136/bmjgh-2021-007074 PMC8559094

[CR31] Bobo, F. T., Asante, A., Woldie, M., & Hayen, A. (2021b). Poor coverage and quality for poor women: Inequalities in quality antenatal care in nine East African countries. *Health Policy and Planning*. 10.1093/heapol/czaa192 33822943 10.1093/heapol/czaa192

[CR32] Bobo, F. T., Yesuf, E. A., & Woldie, M. (2017). Inequities in utilization of reproductive and maternal health services in Ethiopia. *International Journal for Equity in Health*. 10.1186/s12939-017-0602-2 28629358 10.1186/s12939-017-0602-2 PMC5477250

[CR33] Boerma, T., Ronsmans, C., Melesse, D. Y., Barros, A. J., Barros, F. C., Juan, L., Moller, A. B., Say, L., Hosseinpoor, A. R., Yi, M., & Neto, D. D. L. R. (2018). Global epidemiology of use of and disparities in caesarean sections. *The Lancet,**392*(10155), 1341–1348. 10.1016/S0140-6736(18)31928-7 10.1016/S0140-6736(18)31928-7 30322584

[CR34] Buse, K., & Hawkes, S. (2015). Health in the sustainable development goals: Ready for a paradigm shift? *Globalization and Health,**11*(1), 13–13. 10.1186/s12992-015-0098-8 25890267 10.1186/s12992-015-0098-8 PMC4389312

[CR35] Çalışkan, Z., Kılıç, D., Öztürk, S., & Atılgan, E. (2015). Equity in maternal health care service utilization: A systematic review for developing countries. *International Journal of Public Health,**60*(7), 815–825. 26298441 10.1007/s00038-015-0711-x

[CR36] Campbell, O. M. R. P., Calvert, C. P., Testa, A. M., Strehlow, M. M. D., Benova, L. P., Keyes, E. M., Donnay, F. M. D., Macleod, D. M., Gabrysch, S. P., Rong, L. M. M., Ronsmans, C. D., Sadruddin, S. P., Koblinsky, M. P., & Bailey, P. D. (2016). The scale, scope, coverage, and capability of childbirth care. *The Lancet (British Edition),**388*(10056), 2193–2208. 10.1016/S0140-6736(16)31528-8 10.1016/S0140-6736(16)31528-8 27642023

[CR37] Chauhan, B. G., & Jungari, S. (2021). Factors affecting the utilization of maternal and child health care services in tribal dominated population states of India. *International Quarterly Community Health Education*. 10.1177/0272684x20972857 10.1177/0272684X20972857 33201772

[CR38] Chauhan, B. G., & Radkar, A. (2023). Trends and inequalities in caesarean&nbsp;section delivery in India, 1992–2021. *Journal of Public Health (Berl.)*. 10.1007/s10389-023-01888-3

[CR39] Chowdhury, S. S. A., Kundu, S., & Sharif, A. B. (2023). Socioeconomic and geographical inequalities in using skilled birth attendants during delivery in Bangladesh over two decades. *BMC Pregnancy and Childbirth*. 10.1186/s12884-023-05754-8 37296394 10.1186/s12884-023-05754-8 PMC10251570

[CR40] D’haenens, F., Van Rompaey, B., Swinnen, E., Dilles, T., & Beeckman, K. (2020). The effects of continuity of care on the health of mother and child in the postnatal period: A systematic review. *European Journal of Public Health,**30*(4), 749–760. 10.1093/eurpub/ckz082 31121019 10.1093/eurpub/ckz082

[CR41] Daka, D. W., Woldie, M., Ergiba, M. S., Sori, B. K., Bayisa, D. A., Amente, A. B., & Bobo, F. T. (2020). Inequities in the uptake of reproductive and maternal health services in the biggest regional state of Ethiopia: Too far from “leaving no one behind.” *ClinicoEconomics and Outcomes Research*. 10.2147/ceor.s269955 33116699 10.2147/CEOR.S269955 PMC7585815

[CR42] Dankwah, E., Kirychuk, S., Zeng, W., Feng, C., & Farag, M. (2019). Socioeconomic inequalities in the use of caesarean section delivery in Ghana: A cross-sectional study using nationally representative data. *International Journal for Equity in Health*. 10.1186/s12939-019-1063-6 31653255 10.1186/s12939-019-1063-6 PMC6814993

[CR43] Dawson, P., Jaye, C., Gauld, R., & Hay-Smith, J. (2019). Barriers to equitable maternal health in Aotearoa New Zealand: An integrative review. *International Journal for Equity in Health,**18*(1), 168–168. 10.1186/s12939-019-1070-7 31666134 10.1186/s12939-019-1070-7 PMC6822457

[CR44] De La Torre, A., Nikoloski, Z., & Mossialos, E. (2018). Equity of access to maternal health interventions in Brazil and Colombia: A retrospective study. *International Journal for Equity in Health*. 10.1186/s12939-018-0752-x 29642907 10.1186/s12939-018-0752-x PMC5896161

[CR45] Dewau, R., Angaw, D. A., Kassa, G. M., Dagnew, B., Yeshaw, Y., Muche, A., Feleke, D. G., Molla, E., Yehuala, E. D., Tadesse, S. E., Yalew, M., Fentaw, Z., Asfaw, A. H., Andargie, A., Chanie, M. G., Ayele, W. M., Hassen, A. M., Damtie, Y., Hussein, F. M., & Ayele, T. A. (2021). Urban-rural disparities in institutional delivery among women in East Africa: A decomposition analysis. *PLoS ONE*. 10.1371/journal.pone.0255094 34329310 10.1371/journal.pone.0255094 PMC8323938

[CR46] Do, M., Soelaeman, R., & Hotchkiss, D. R. (2015). Explaining inequity in the use of institutional delivery services in selected countries. *Maternal and Child Health Journal*. 10.1007/s10995-014-1561-5 24985698 10.1007/s10995-014-1561-5

[CR47] Ekholuenetale, M., Nzoputam, C. I., & Barrow, A. (2021). Prevalence and socioeconomic inequalities in eight or more antenatal care contacts in Ghana: Findings from 2019 population-based data. *International Journal of Women’s Health*. 10.2147/ijwh.s306302 33790659 10.2147/IJWH.S306302 PMC8001171

[CR48] Fagbamigbe, A. F., & Oyedele, O. K. (2022). Multivariate decomposition of trends, inequalities and predictors of skilled birth attendants utilisation in Nigeria (1990–2018): A cross-sectional analysis of change drivers. *British Medical Journal Open*. 10.1136/bmjopen-2021-051791 10.1136/bmjopen-2021-051791 PMC8981288 35379613

[CR49] Fan, X., Zhou, Z., Dang, S., Xu, Y., Gao, J., Zhou, Z., Su, M., Wang, D., & Chen, G. (2017). Exploring status and determinants of prenatal and postnatal visits in western China: In the background of the new health system reform. *BMC Public Health*. 10.1186/s12889-017-4601-4 28728550 10.1186/s12889-017-4601-4 PMC5520235

[CR50] Flores, T. R., Neves, R. G., Mielke, G. I., Bertoldi, A. D., & Nunes, B. P. (2021). Inequalities on coverage of prenatal assistance in Brazil: A nationwide study. *Ciência & Saude Coletiva*. 10.1590/1413-81232021262.26792019 10.1590/1413-81232021262.26792019 33605336

[CR51] Fonseca, S. C., Carvalho, Z. S. B. D., Kale, P. L., Boschi-Pinto, C., & Guimarães, J. C. C. (2022). Trends in sociodemographic inequalities in prenatal care in Baixada Litorânea, a region of the state of Rio de Janeiro, Brazil, 2000–2020: An ecological study. *Epidemiologia e Serviços De Saúde*. 10.1590/s2237-96222022000300006 36351059 10.1590/S2237-96222022000300006 PMC9887988

[CR52] França, G. V. A., Restrepo-Méndez, M. C., Maia, M. F. S., Victora, C. G., & Barros, A. J. D. (2016). Coverage and equity in reproductive and maternal health interventions in Brazil: Impressive progress following the implementation of the Unified Health System. *International Journal for Equity in Health*. 10.1186/s12939-016-0445-2 27852276 10.1186/s12939-016-0445-2 PMC5112713

[CR53] Gandhi, S., Dash, U., & Suresh Babu, M. (2022). Horizontal inequity in the utilisation of Continuum of Maternal Health care Services (CMHS) in India: An investigation of ten years of National Rural Health Mission (NRHM). *International Journal for Equity in Health*. 10.1186/s12939-021-01602-3 35033087 10.1186/s12939-021-01602-3 PMC8760767

[CR54] Gandhi, S., Maharatha, T. M., Dash, U., & Babu, M. S. (2021). Level of inequality and the role of governance indicators in the coverage of reproductive maternal and child healthcare services: Findings from India. *PLoS ONE*. 10.1371/journal.pone.0258244 34767556 10.1371/journal.pone.0258244 PMC8589169

[CR55] Gebre, E., Worku, A., & Bukola, F. (2018). Inequities in maternal health services utilization in Ethiopia 2000–2016: Magnitude, trends, and determinants. *Reproductive Health*. 10.1186/s12978-018-0556-x 29973244 10.1186/s12978-018-0556-x PMC6031117

[CR56] Godha, D., & Hotchkiss, D. R. (2022). A decade of conditional cash transfer programs for reproductive health in India: How did equality fare? *BMC Public Health*. 10.1186/s12889-022-12563-9 35216569 10.1186/s12889-022-12563-9 PMC8876831

[CR57] Goli, S., Nawal, D., Rammohan, A., Sekher, T. V., & Singh, D. (2018). Decomposing the socioeconomic inequality in utilization of maternal health care services in selected countries of South Asia and Sub-Saharan Africa. *Journal of Biosocial Science*. 10.1017/s0021932017000530 29081310 10.1017/S0021932017000530

[CR58] Guilmoto, C. Z., & Dumont, A. (2019). Trends, regional variations, and socioeconomic disparities in Cesarean Births in India, 2010–2016. *JAMA Network Open*. 10.1001/jamanetworkopen.2019.0526 30901040 10.1001/jamanetworkopen.2019.0526 PMC6583298

[CR59] Hernández-Vásquez, A., Chacón-Torrico, H., & Bendezu-Quispe, G. (2022). Geographic and socioeconomic inequalities in cesarean birth rates in Peru: A comparison between 2009 and 2018. *Birth*. 10.1111/birt.12572 34240458 10.1111/birt.12572

[CR60] Himanshu, M., & Källestål, C. (2017). Regional inequity in complete antenatal services and public emergency obstetric care is associated with greater burden of maternal deaths: Analysis from consecutive district level facility survey of Karnataka India. *International Journal for Equity in Health*. 10.1186/s12939-017-0573-3 28490355 10.1186/s12939-017-0573-3 PMC5426006

[CR61] Hodge, A., Firth, S., Bermejo, R., 3rd., Zeck, W., & Jimenez-Soto, E. (2016). Utilisation of health services and the poor: Deconstructing wealth-based differences in facility-based delivery in the Philippines. *BMC Public Health*. 10.1186/s12889-016-3148-0 27383189 10.1186/s12889-016-3148-0 PMC4936303

[CR62] Hosseinpoor, A. R., Bergen, N., Barros, A. J. D., Wong, K. L. M., Boerma, T., & Victora, C. G. (2016). Monitoring subnational regional inequalities in health: Measurement approaches and challenges. *International Journal for Equity in Health*. 10.1186/s12939-016-0307-y 26822991 10.1186/s12939-016-0307-y PMC4730638

[CR63] Huda, T. M., Hayes, A., & Dibley, M. J. (2018). Examining horizontal inequity and social determinants of inequality in facility delivery services in three South Asian countries. *Journal of Global Health*. 10.7189/jogh.08.010416 29977529 10.7189/jogh.08.010416 PMC6008508

[CR64] Jalloh, M. B., Bah, A. J., James, P. B., Sevalie, S., Hann, K., & Shmueli, A. (2019). Impact of the free healthcare initiative on wealth-related inequity in the utilization of maternal & child health services in Sierra Leone. *BMC Health Services Research*. 10.1186/s12913-019-4181-3 31159785 10.1186/s12913-019-4181-3 PMC6547484

[CR65] Jongh, T. E., Gurol-Urganci, I., Allen, E., Jiayue Zhu, N., & Atun, R. (2016). Barriers and enablers to integrating maternal and child health services to antenatal care in low and middle income countries. *BJOG an International Journal of Obstetrics and Gynaecology,**123*(4), 549–557. 10.1111/1471-0528.13898 26861695 10.1111/1471-0528.13898 PMC4768640

[CR66] Kachoria, A. G., Mubarak, M. Y., Singh, A. K., Somers, R., Shah, S., & Wagner, A. L. (2022). The association of religion with maternal and child health outcomes in South Asian countries. *PLoS ONE,**17*(7), Article e0271165. 10.1371/journal.pone.0271165 35819940 10.1371/journal.pone.0271165 PMC9275688

[CR67] Kakama, A. A., & Basaza, R. (2022). Trends in inequality in maternal and child health and health care in Uganda: Analysis of the Uganda demographic and health surveys. *BMC Health Services Research*. 10.1186/s12913-022-08630-x 36266643 10.1186/s12913-022-08630-x PMC9585693

[CR68] Kamal, N., Curtis, S., Hasan, M. S., & Jamil, K. (2016). Trends in equity in use of maternal health services in urban and rural Bangladesh. *International Journal for Equity in Health*. 10.1186/s12939-016-0311-2 26883742 10.1186/s12939-016-0311-2 PMC4756462

[CR69] Keats, E. C., Akseer, N., Bhatti, Z., Macharia, W., Ngugi, A., Rizvi, A., & Bhutta, Z. A. (2018). Assessment of inequalities in coverage of essential reproductive, maternal, newborn, child, and adolescent health interventions in Kenya. *JAMA Network Open*. 10.1001/jamanetworkopen.2018.5152 30646326 10.1001/jamanetworkopen.2018.5152 PMC6324360

[CR70] Khadr, Z. (2020). Monitoring the decomposition of wealth-related inequality in the use of regular antenatal care in Egypt (1995–2014). *BMC Public Health*. 10.1186/s12889-020-09412-y 32854669 10.1186/s12889-020-09412-y PMC7453517

[CR71] Khan, M. N., Islam, M. M., & Rahman, M. M. (2018). Inequality in utilization of cesarean delivery in Bangladesh: A decomposition analysis using nationally representative data. *Public Health*. 10.1016/j.puhe.2018.01.015 29518616 10.1016/j.puhe.2018.01.015

[CR72] Kien, V. D., Jat, T. R., Phu, T. V., Cuong, L. M., Anh, V. T. M., Chu, N. V., Duong, T. T., Long, V. H., & Dung, T. C. (2019). Trends in socioeconomic inequalities in the use of antenatal care services by women aged 15 to 49 years in Vietnam. *Asia Pacific Journal of Public Health*. 10.1177/1010539519857305 31232081 10.1177/1010539519857305

[CR73] Kim, C., Saeed, K. M. A., Salehi, A. S., & Zeng, W. (2016). An equity analysis of utilization of health services in Afghanistan using a national household survey. *BMC Public Health*. 10.1186/s12889-016-3894-z 27919238 10.1186/s12889-016-3894-z PMC5139141

[CR74] Kim, M. K., Kim, S. A., Oh, J., Kim, C. E., & Arsenault, C. (2022). Measuring effective coverage of maternal and child health services in Cambodia: A retrospective analysis of demographic and health surveys from 2005 to 2014. *British Medical Journal Open*. 10.1136/bmjopen-2022-062028 10.1136/bmjopen-2022-062028 PMC9454061 36691182

[CR75] Kitila, S. B., Feyissa, G. T., Olika, A. K., & Wordofa, M. A. (2022). Maternal healthcare in low- and middle-income countries: A scoping review. *Health Services Insights,**15*, 11786329221100310–11786329221100310. 10.1177/11786329221100310 35615600 10.1177/11786329221100310 PMC9125054

[CR76] Kpodotsi, A., Baku, E. A., Adams, J. H., & Alaba, O. (2021). Socioeconomic inequalities in access and use of skilled birth attendants during childbirth in Ghana: A decomposition analysis. *BMC Pregnancy and Childbirth*. 10.1186/s12884-021-04290-7 34969366 10.1186/s12884-021-04290-7 PMC8719398

[CR77] Krieger, N. (2012). Methods for the scientific study of discrimination and health: An ecosocial approach. *American Journal of Public Health (1971),**102*(5), 936–945. 10.2105/ajph.2011.300544 10.2105/AJPH.2011.300544 PMC3484783 22420803

[CR78] Krishnamoorthy, Y., Majella, M. G., & Rajaa, S. (2020). Equity in coverage of maternal and newborn care in India: Evidence from a nationally representative survey. *Health Policy and Planning*. 10.1093/heapol/czaa020 32236550 10.1093/heapol/czaa020

[CR79] Kumar, G., Choudhary, T. S., Srivastava, A., Upadhyay, R. P., Taneja, S., Bahl, R., Martines, J., Bhan, M. K., Bhandari, N., & Mazumder, S. (2019). Utilisation, equity and determinants of full antenatal care in India: Analysis from the national family health survey 4. *BMC Pregnancy and Childbirth*. 10.1186/s12884-019-2473-6 31488080 10.1186/s12884-019-2473-6 PMC6727513

[CR80] Lam, N. D., Anh, N. D., Ha, N. T. T., Vinh, T. Q., Anh, V. T. M., & Kien, V. D. (2019). Socioeconomic inequalities in post-natal health checks for the newborn in Vietnam. *International Journal for Equity in Health*. 10.1186/s12939-019-1029-8 31420044 10.1186/s12939-019-1029-8 PMC6697903

[CR81] Langlois, E. V., Miszkurka, M., Zunzunegui, M. V., Ghaffar, A., Ziegler, D., & Karp, I. (2015). Inequities in postnatal care in low- and middle-income countries: A systematic review and meta-analysis. *Bulletin of the World Health Organization,**93*(4), 259–270. 10.2471/BLT.14.140996 26229190 10.2471/BLT.14.140996 PMC4431556

[CR82] Leventhal, D. G. P., Crochemore-Silva, I., Vidaletti, L. P., Armenta-Paulino, N., Barros, A. J. D., & Victora, C. G. (2021). Delivery channels and socioeconomic inequalities in coverage of reproductive, maternal, newborn, and child health interventions: Analysis of 36 cross-sectional surveys in low-income and middle-income countries. *The Lancet Global Health*. 10.1016/s2214-109x(21)00204-7 34051180 10.1016/S2214-109X(21)00204-7 PMC8295042

[CR83] Liang, H., Fang, S., Liu, S., Liu, X., Li, Y., Li, M., & Zhang, Y. (2017). Equity in maternal- and infant-care services in China: A trend analysis based on residence and area (2000–2014). *International Journal of Health Planning and Management*. 10.1002/hpm.2438 28707333 10.1002/hpm.2438

[CR84] Lohela, T. J., Nesbitt, R. C., Pekkanen, J., & Gabrysch, S. (2019). Comparing socioeconomic inequalities between early neonatal mortality and facility delivery: Cross-sectional data from 72 low- and middle-income countries. *Scientific Reports*. 10.1038/s41598-019-45148-5 31278283 10.1038/s41598-019-45148-5 PMC6611781

[CR85] Lukwa, A. T., Siya, A., Odunitan-Wayas, F. A., & Alaba, O. (2022). Decomposing maternal socioeconomic inequalities in Zimbabwe; leaving no woman behind. *BMC Pregnancy and Childbirth*. 10.1186/s12884-022-04571-9 35321687 10.1186/s12884-022-04571-9 PMC8944016

[CR86] Mallmann, M. B., Boing, A. F., Tomasi, Y. T., Anjos, J. C. D., & Boing, A. C. (2018). Evolution of socioeconomic inequalities in conducting prenatal consultations among Brazilian parturient women: Analysis of the period 2000–2015. *Epidemiologia e Serviços De Saúde*. 10.5123/s1679-49742018000400014 30517352 10.5123/S1679-49742018000400014

[CR87] Mangham-Jefferies, L., Pitt, C., Cousens, S., Mills, A., & Schellenberg, J. (2014). Cost-effectiveness of strategies to improve the utilization and provision of maternal and newborn health care in low-income and lower-middle-income countries: A systematic review. *BMC Pregnancy and Childbirth,**14*(1), 243–243. 10.1186/1471-2393-14-243 25052536 10.1186/1471-2393-14-243 PMC4223592

[CR88] McCartney, G., Collins, C., & Mackenzie, M. (2013). What (or who) causes health inequalities: Theories, evidence and implications? *Health Policy (Amsterdam),**113*(3), 221–227. 10.1016/j.healthpol.2013.05.021 10.1016/j.healthpol.2013.05.021 23810172

[CR89] McKinnon, B., Harper, S., & Kaufman, J. S. (2016). Do socioeconomic inequalities in neonatal mortality reflect inequalities in coverage of maternal health services? Evidence from 48 low- and middle-income countries. *Maternal and Child Health Journal*. 10.1007/s10995-015-1841-8 26546016 10.1007/s10995-015-1841-8

[CR90] Mehata, S., Paudel, Y. R., Dariang, M., Aryal, K. K., Lal, B. K., Khanal, M. N., & Thomas, D. (2017). Trends and inequalities in use of maternal health care services in Nepal: Strategy in the search for improvements. *BioMed Research International*. 10.1155/2017/5079234 28808658 10.1155/2017/5079234 PMC5541802

[CR91] Mekonnen, T., Dune, T., & Perz, J. (2019). Maternal health service utilisation of adolescent women in sub-Saharan Africa: A systematic scoping review. *BMC Pregnancy and Childbirth,**19*(1), 366–366. 10.1186/s12884-019-2501-6 31638927 10.1186/s12884-019-2501-6 PMC6805384

[CR92] Memirie, S. T., Verguet, S., Norheim, O. F., Levin, C., & Johansson, K. A. (2016). Inequalities in utilization of maternal and child health services in Ethiopia: The role of primary health care. *BMC Health Services Research*. 10.1186/s12913-016-1296-7 26867540 10.1186/s12913-016-1296-7 PMC4751648

[CR93] Mezmur, M., Navaneetham, K., Letamo, G., & Bariagaber, H. (2017). Socioeconomic inequalities in the uptake of maternal healthcare services in Ethiopia. *BMC Health Services Research*. 10.1186/s12913-017-2298-9 28532407 10.1186/s12913-017-2298-9 PMC5441003

[CR94] Mishra, P. S., Pautunthang, N., & Marbaniang, S. P. (2021). Geographical divide led inequality in accessing maternal healthcare services between hills and valley regions of Manipur state, India. *Clinical Epidemiology and Global Health*. 10.1016/j.cegh.2021.10074434179566

[CR95] Misu, F., & Alam, K. (2023a). Comparison of inequality in utilization of maternal healthcare services between Bangladesh and Pakistan: Evidence from the demographic health survey 2017–2018. *Reproductive Health*. 10.1186/s12978-023-01595-y 36915151 10.1186/s12978-023-01595-y PMC10009948

[CR96] Misu, F., & Alam, K. (2023b). Comparison of inequality in utilization of postnatal care services between Bangladesh and Pakistan: Evidence from the Demographic and Health Survey 2017–2018. *BMC Pregnancy and Childbirth*. 10.1186/s12884-023-05778-0 37349680 10.1186/s12884-023-05778-0 PMC10286509

[CR97] Morgan, J., & Breau, M. G. (2024). Access to maternal health services for Indigenous women in low- and middle-income countries: An updated integrative review of the literature from 2018 to 2023. *Rural and Remote Health,**24*(2), 1–12. 10.22605/RRH8520 10.22605/RRH8520 38826130

[CR98] Nababan, H. Y., Hasan, M., Marthias, T., Dhital, R., Rahman, A., & Anwar, I. (2017). Trends and inequities in use of maternal health care services in Indonesia, 1986–2012. *International Journal of Women’s Health*. 10.2147/ijwh.s144828 29343991 10.2147/IJWH.S144828 PMC5749568

[CR99] Nguyen, P. T., Rahman, M. S., Le, P. M., Nguyen, H. V., Vu, K. D., Nguyen, H. L., Dao, A. T. M., Khuong, L. Q., Hoang, M. V., & Gilmour, S. (2021). Trends in, projections of, and inequalities in reproductive, maternal, newborn and child health service coverage in Vietnam 2000–2030: A Bayesian analysis at national and sub-national levels. *The Lancet Regional Health Western Pacific*. 10.1016/j.lanwpc.2021.100230 34528011 10.1016/j.lanwpc.2021.100230 PMC8342952

[CR100] Novignon, J., Ofori, B., Tabiri, K. G., & Pulok, M. H. (2019). Socioeconomic inequalities in maternal health care utilization in Ghana. *International Journal for Equity in Health*. 10.1186/s12939-019-1043-x 31488160 10.1186/s12939-019-1043-x PMC6729067

[CR101] Nwosu, C. O., & Ataguba, J. E. (2019). Socioeconomic inequalities in maternal health service utilisation: A case of antenatal care in Nigeria using a decomposition approach. *BMC Public Health*. 10.1186/s12889-019-7840-8 31703734 10.1186/s12889-019-7840-8 PMC6842188

[CR102] O’Donnell, O., van Doorslaer, E., Wagstaff, A., Lindelow, M., & World, B. (2008). Analyzing health equity using household survey data: A guide to techniques and their implementation. *World Bank*. 10.1596/978-0-8213-6933-3

[CR103] Ogundele, O. J., Pavlova, M., & Groot, W. (2018). Examining trends in inequality in the use of reproductive health care services in Ghana and Nigeria. *BMC Pregnancy and Childbirth*. 10.1186/s12884-018-2102-9 30545328 10.1186/s12884-018-2102-9 PMC6293518

[CR104] Ogundele, O. J., Pavlova, M., & Groot, W. (2020). Inequalities in reproductive health care use in five West-African countries: A decomposition analysis of the wealth-based gaps. *International Journal for Equity in Health*. 10.1186/s12939-020-01167-7 32220250 10.1186/s12939-020-01167-7 PMC7099835

[CR105] Okoli, C., Hajizadeh, M., Rahman, M. M., & Khanam, R. (2020). Geographical and socioeconomic inequalities in the utilization of maternal healthcare services in Nigeria: 2003–2017. *BMC Health Services Research*. 10.1186/s12913-020-05700-w 32912213 10.1186/s12913-020-05700-w PMC7488161

[CR106] Okyere, J., Duah, H. O., Seidu, A.-A., Ahinkorah, B. O., & Budu, E. (2022). Inequalities in prevalence of birth by caesarean section in Ghana from 1998–2014. *BMC Pregnancy and Childbirth*. 10.1186/s12884-022-04378-8 35065625 10.1186/s12884-022-04378-8 PMC8783997

[CR107] Onarheim, K. H., Taddesse, M., Norheim, O. F., Abdullah, M., & Miljeteig, I. (2015). Towards universal health coverage for reproductive health services in Ethiopia: Two policy recommendations. *International Journal for Equity in Health*. 10.1186/s12939-015-0218-3 26419910 10.1186/s12939-015-0218-3 PMC4588686

[CR108] Panda, B. K., Kumar, G., & Awasthi, A. (2020a). District level inequality in reproductive, maternal, neonatal and child health coverage in India. *BMC Public Health*. 10.1186/s12889-020-8151-9 31937270 10.1186/s12889-020-8151-9 PMC6961337

[CR109] Panda, B. K., Nayak, I., & Mishra, U. S. (2020b). Determinant of inequality in cesarean delivery in India: A decomposition analysis. *Health Care for Women International*. 10.1080/07399332.2020.1711757 31928373 10.1080/07399332.2020.1711757

[CR110] Pandey, A. R., Ojha, B., Shrestha, N., Maskey, J., Sharma, D., Godwin, P., Chalise, B., & Aryal, K. K. (2021). Progress in reducing inequalities in reproductive, maternal, newborn and child health services in Nepal. *Journal of Nepal Health Research Council*. 10.33314/jnhrc.v19i1.3375 33934149 10.33314/jnhrc.v19i1.3375

[CR111] Paredes, K. P. P. (2016). Inequality in the use of maternal and child health services in the Philippines: Do pro-poor health policies result in more equitable use of services? *International Journal for Equity in Health*. 10.1186/s12939-016-0473-y 27832778 10.1186/s12939-016-0473-y PMC5105289

[CR112] Paul, S. (2021). Are the poor catching up with the rich in utilising maternal health care services? Evidence from India. *Journal of Health Management*. 10.1177/09720634211035212

[CR113] Puchalski Ritchie, L. M., Khan, S., Moore, J. E., Timmings, C., van Lettow, M., Vogel, J. P., Khan, D. N., Mbaruku, G., Mrisho, M., Mugerwa, K., Uka, S., Gülmezoglu, A. M., & Straus, S. E. (2016). Low- and middle-income countries face many common barriers to implementation of maternal health evidence products. *Journal of Clinical Epidemiology,**76*, 229–237. 10.1016/j.jclinepi.2016.02.017 26931284 10.1016/j.jclinepi.2016.02.017

[CR114] Pulok, M. H., Chirwa, G. C., Novignon, J., Aizawa, T., & Makate, M. (2020). Levels of and changes in socioeconomic inequality in delivery care service: A decomposition analysis using Bangladesh Demographic Health Surveys. *PLoS ONE*. 10.1371/journal.pone.0242325 33253221 10.1371/journal.pone.0242325 PMC7703934

[CR115] Pulok, M. H., Sabah, M.N.-U., Uddin, J., & Enemark, U. (2016). Progress in the utilization of antenatal and delivery care services in Bangladesh: Where does the equity gap lie? *BMC Pregnancy and Childbirth*. 10.1186/s12884-016-0970-4 27473150 10.1186/s12884-016-0970-4 PMC4967314

[CR116] Pulok, M. H., Uddin, J., Enemark, U., & Hossin, M. Z. (2018a). Socioeconomic inequality in maternal healthcare: An analysis of regional variation in Bangladesh. *Health & Place*. 10.1016/j.healthplace.2018.06.004 10.1016/j.healthplace.2018.06.004 29960144

[CR117] Pulok, M. H., Uddin, J., Enemark, U., & Hossin, M. Z. (2018b). Socioeconomic inequality in maternal healthcare: An analysis of regional variation in Bangladesh. *Health & Place,**52*, 205–214. 29960144 10.1016/j.healthplace.2018.06.004

[CR118] Quizhpe, E., Sebastian, M. S., Teran, E., & Pulkki-Brännström, A.-M. (2020). Socioeconomic inequalities in women’s access to health care: Has Ecuadorian health reform been successful? *International Journal for Equity in Health*. 10.1186/s12939-020-01294-1 33036631 10.1186/s12939-020-01294-1 PMC7545545

[CR119] Rahman, A., Nisha, M. K., Begum, T., Ahmed, S., Alam, N., & Anwar, I. (2017a). Trends, determinants and inequities of 4(+) ANC utilisation in Bangladesh. *Journal of Health, Population and Nutrition,*. 10.1186/s41043-016-0078-5 28086970 10.1186/s41043-016-0078-5 PMC5237328

[CR120] Rahman, M. M., Karan, A., Rahman, M. S., Parsons, A., Abe, S. K., Bilano, V., Awan, R., Gilmour, S., & Shibuya, K. (2017b). Progress toward universal health coverage: A comparative analysis in 5 South Asian countries. *JAMA Internal Medicine*. 10.1001/jamainternmed.2017.3133 28759681 10.1001/jamainternmed.2017.3133 PMC5710570

[CR121] Rahman, M. A., Kundu, S., Rashid, H. O., Shanto, H. H., Rahman, M. M., Khan, B., Howlader, M. H., & Islam, M. A. (2022a). Socioeconomic inequalities in utilizing facility delivery in Bangladesh: A decomposition analysis using nationwide 2017–2018 demographic and health survey data. *PLoS ONE*. 10.1371/journal.pone.0278093 36441796 10.1371/journal.pone.0278093 PMC9704681

[CR122] Rahman, M. A., Sultana, S., Kundu, S., Islam, M. A., Roshid, H. O., Khan, Z. I., Tohan, M., Jahan, N., Khan, B., & Howlader, M. H. (2022b). Trends and patterns of inequalities in using facility delivery among reproductive-age women in Bangladesh: A decomposition analysis of 2007–2017 Demographic and Health Survey data. *British Medical Journal Open*. 10.1136/bmjopen-2022-065674 10.1136/bmjopen-2022-065674 PMC9806084 36581408

[CR123] Ravit, M., Audibert, M., Ridde, V., De Loenzien, M., Schantz, C., & Dumont, A. (2018). Do free caesarean section policies increase inequalities in Benin and Mali? *International Journal for Equity in Health*. 10.1186/s12939-018-0789-x 29871645 10.1186/s12939-018-0789-x PMC5989420

[CR124] Reynolds, H. W., Emelita, L. W., & Heidi, T. (2006). Adolescents’ use of maternal and child health services in developing countries. *International Family Planning Perspectives,**32*(1), 6–16. 10.1363/3200606 16723297 10.1363/3200606

[CR125] Rios Quituizaca, P., Gatica-Domínguez, G., Nambiar, D., Ferreira Santos, J. L., Brück, S., Vidaletti Ruas, L., & Barros, A. J. D. (2021). National and subnational coverage and inequalities in reproductive, maternal, newborn, child, and sanitary health interventions in Ecuador: A comparative study between 1994 and 2012. *International Journal for Equity in Health*. 10.1186/s12939-020-01359-1 33509210 10.1186/s12939-020-01359-1 PMC7842066

[CR126] Rios-Quituizaca, P., Gatica-Domínguez, G., Nambiar, D., Santos, J. L. F., & Barros, A. J. D. (2022). Ethnic inequalities in reproductive, maternal, newborn and child health interventions in Ecuador: A study of the 2004 and 2012 national surveys. *EClinicalMedicine*. 10.1016/j.eclinm.2022.101322 35284805 10.1016/j.eclinm.2022.101322 PMC8904232

[CR127] Sanogo, N. A., & Yaya, S. (2020). Wealth status, health insurance, and maternal health care utilization in Africa: Evidence from gabon. *BioMed Research International*. 10.1155/2020/4036830 32461984 10.1155/2020/4036830 PMC7212326

[CR128] Sapkota, V. P., Bhusal, U. P., & Acharya, K. (2021). Trends in national and subnational wealth related inequalities in use of maternal health care services in Nepal: An analysis using demographic and health surveys (2001–2016). *BMC Public Health*. 10.1186/s12889-020-10066-z 33397359 10.1186/s12889-020-10066-z PMC7784024

[CR171] Sarikhani, Y., Najibi, S. M., & Razavi, Z. (2024). Key barriers to the provision and utilization of maternal health services in low-and lower-middle-income countries; a scoping review. *BMC Women's Health,**24*(1), 1–15. 10.1186/s12905-024-03177-x 38840156 10.1186/s12905-024-03177-x PMC11151574

[CR129] Seidu, A.-A., Okyere, J., Budu, E., Duah, H. O., & Ahinkorah, B. O. (2022). Inequalities in antenatal care in Ghana, 1998–2014. *BMC Pregnancy and Childbirth*. 10.1186/s12884-022-04803-y 35698085 10.1186/s12884-022-04803-y PMC9190076

[CR130] Selebano, K. M., & Ataguba, J. E. (2021). Decomposing socio-economic inequalities in antenatal care utilisation in 12 Southern African Development Community countries. *SSM - Population Health*. 10.1016/j.ssmph.2021.101004 34988282 10.1016/j.ssmph.2021.101004 PMC8703074

[CR131] Shibre, G., Idriss-Wheeler, D., Bishwajit, G., & Yaya, S. (2020a). Observed trends in the magnitude of socioeconomic and area-based inequalities in use of caesarean section in Ethiopia: A cross-sectional study. *BMC Public Health*. 10.1186/s12889-020-09297-x 32781997 10.1186/s12889-020-09297-x PMC7418379

[CR132] Shibre, G., Mekonnen, W., & Haile Mariam, D. (2023). Decomposition analysis of women’s empowerment-based inequalities in the use of maternal health care services in Ethiopia: Evidence from demographic and health surveys. *PLoS ONE*. 10.1371/journal.pone.0285024 37104524 10.1371/journal.pone.0285024 PMC10138853

[CR133] Shibre, G., Zegeye, B., Ahinkorah, B. O., Idriss-Wheeler, D., Keetile, M., & Yaya, S. (2021). Sub-regional disparities in the use of antenatal care service in Mauritania: Findings from nationally representative demographic and health surveys (2011–2015). *BMC Public Health*. 10.1186/s12889-021-11836-z 34627186 10.1186/s12889-021-11836-z PMC8501590

[CR134] Shibre, G., Zegeye, B., Ahinkorah, B. O., Keetile, M., & Yaya, S. (2020b). Magnitude and trends in socio-economic and geographic inequality in access to birth by cesarean section in Tanzania: Evidence from five rounds of Tanzania demographic and health surveys (1996–2015). *Archives of Public Health*. 10.1186/s13690-020-00466-3 32944238 10.1186/s13690-020-00466-3 PMC7491176

[CR135] Shibre, G., Zegeye, B., Idriss-Wheeler, D., Ahinkorah, B. O., Oladimeji, O., & Yaya, S. (2020c). Socioeconomic and geographic variations in antenatal care coverage in Angola: Further analysis of the 2015 demographic and health survey. *BMC Public Health*. 10.1186/s12889-020-09320-1 32799833 10.1186/s12889-020-09320-1 PMC7429730

[CR136] Shirisha, P., Vaidyanathan, G., & Muraleedharan, V. R. (2022). Are the poor catching up with the rich in utilising reproductive, maternal, new born and child health services: An application of delivery channels framework in Indian context. *Journal of Health Management*. 10.1177/09720634221079071

[CR137] Shreezal, G. C., & Adhikari, N. (2023). Decomposing inequality in maternal and child health (MCH) services in Nepal. *BMC Public Health*. 10.1186/s12889-023-15906-2 10.1186/s12889-023-15906-2 PMC10226207 37248553

[CR138] Silva, I. C. M. D., Restrepo-Mendez, M. C., Costa, J. C., Ewerling, F., Hellwig, F., Ferreira, L. Z., Ruas, L. P. V., Joseph, G., & Barros, A. J. (2018). Measurement of social inequalities in health: Concepts and methodological approaches in the Brazilian context. *Epidemiologia e Serviços De Saúde*. 10.5123/s1679-49742018000100017 29513856 10.5123/S1679-49742018000100017 PMC7705122

[CR139] Sk, M. I. K., Ali, B., Biswas, M. M., & Saha, M. K. (2022). Disparities in three critical maternal health indicators amongst Muslims: Vis-a-vis the results reflected on National Health Mission. *BMC Public Health*. 10.1186/s12889-022-12662-7 35139830 10.1186/s12889-022-12662-7 PMC8830117

[CR140] Souza, J. P., Day, L. T., Rezende-Gomes, A. C., Zhang, J., Mori, R., Baguiya, A., Jayaratne, K., Osoti, A., Vogel, J. P., Campbell, O., Mugerwa, K. Y., Lumbiganon, P., Tunçalp, Ö., Cresswell, J., Say, L., Moran, A. C., & Oladapo, O. T. (2024). A global analysis of the determinants of maternal health and transitions in maternal mortality. *The Lancet Global Health,**12*(2), e306–e316. 10.1016/S2214-109X(23)00468-0 38070536 10.1016/S2214-109X(23)00468-0

[CR141] Taleb El Hassen, M. V., Cabases, J. M., Zine Eddine El Idrissi, M. D., & Mills, S. (2022). Changes in inequality in use of maternal health care services: Evidence from skilled birth attendance in Mauritania for the period 2007–2015. *International Journal of Environmental Research and Public Health*. 10.3390/ijerph19063566 35329257 10.3390/ijerph19063566 PMC8948710

[CR142] Tangcharoensathien, V., Mills, A., & Palu, T. (2015). Accelerating health equity: The key role of universal health coverage in the sustainable development goals. *BMC Medicine,**13*(1), 101–101. 10.1186/s12916-015-0342-3 25925656 10.1186/s12916-015-0342-3 PMC4415234

[CR143] Tarekegn, W., Tsegaye, S., & Berhane, Y. (2022). Skilled birth attendant utilization trends, determinant and inequality gaps in Ethiopia. *BMC Women’s Health*. 10.1186/s12905-022-01995-5 36419061 10.1186/s12905-022-01995-5 PMC9682649

[CR144] Tesfaye, B., Mathewos, T., & Kebede, M. (2017). Skilled delivery inequality in Ethiopia: To what extent are the poorest and uneducated mothers benefiting? *International Journal for Equity in Health*. 10.1186/s12939-017-0579-x 28511657 10.1186/s12939-017-0579-x PMC5434546

[CR145] Tetteh, J. K., Ameyaw, E. K., Adu, C., Agbaglo, E., Agbadi, P., & Nutor, J. J. (2023). Inequalities in the prevalence of skilled birth attendance in Ghana between 1993 and 2014. *International Health*. 10.1093/inthealth/ihac071 36349614 10.1093/inthealth/ihac071 PMC9977246

[CR146] Thapa, J., Budhathoki, S. S., Gurung, R., Paudel, P., Jha, B., Ghimire, A., Wrammert, J., & Kc, A. (2020). Equity and coverage in the continuum of reproductive, maternal, newborn and child health services in Nepal-projecting the estimates on death averted using the LiST tool. *Maternal and Child Health Journal*. 10.1007/s10995-019-02828-y 31786722 10.1007/s10995-019-02828-y PMC7048704

[CR147] Tricco, A. C., Lillie, E., Zarin, W., O’Brien, K. K., Colquhoun, H., Levac, D., Moher, D., Peters, M. D., Horsley, T., & Weeks, L. (2018). PRISMA extension for scoping reviews (PRISMA-ScR): Checklist and explanation. *Annals of Internal Medicine,**169*(7), 467–473. 30178033 10.7326/M18-0850

[CR148] Tsawe, M., & Susuman, A. S. (2022). Inequalities in maternal healthcare use in Sierra Leone: Evidence from the 2008–2019 Demographic and Health Surveys. *PLoS ONE*. 10.1371/journal.pone.0276102 36228021 10.1371/journal.pone.0276102 PMC9560049

[CR149] Tsegaye, S., Yibeltal, K., Zelealem, H., Worku, W., Demissie, M., Worku, A., & Berhane, Y. (2022). The unfinished agenda and inequality gaps in antenatal care coverage in Ethiopia. *BMC Pregnancy and Childbirth*. 10.1186/s12884-021-04326-y 35093008 10.1186/s12884-021-04326-y PMC8801127

[CR150] Ushie, B. A., Udoh, E. E., & Ajayi, A. I. (2019). Examining inequalities in access to delivery by caesarean section in Nigeria. *PLoS ONE*. 10.1371/journal.pone.0221778 31465505 10.1371/journal.pone.0221778 PMC6715280

[CR151] Vellakkal, S., Gupta, A., Khan, Z., Stuckler, D., Reeves, A., Ebrahim, S., Bowling, A., & Doyle, P. (2017). Has India’s national rural health mission reduced inequities in maternal health services? A pre-post repeated cross-sectional study. *Health Policy and Planning*. 10.1093/heapol/czw100 27515405 10.1093/heapol/czw100 PMC5886191

[CR152] Wabiri, N., Chersich, M., Shisana, O., Blaauw, D., Rees, H., & Dwane, N. (2016). Growing inequities in maternal health in South Africa: A comparison of serial national household surveys. *BMC Pregnancy and Childbirth*. 10.1186/s12884-016-1048-z 27581489 10.1186/s12884-016-1048-z PMC5007803

[CR153] Ward, Z. J., Atun, R., King, G., Sequeira Dmello, B., & Goldie, S. J. (2023). A simulation-based comparative effectiveness analysis of policies to improve global maternal health outcomes. *Nature Medicine,**29*(5), 1262–1272. 10.1038/s41591-023-02311-w 37081227 10.1038/s41591-023-02311-w PMC10202805

[CR154] Wehrmeister, F. C., Barros, A. J. D., Hosseinpoor, A. R., Boerma, T., & Victora, C. G. (2020). Measuring universal health coverage in reproductive, maternal, newborn and child health: An update of the composite coverage index. *PLoS ONE*. 10.1371/journal.pone.0232350 32348356 10.1371/journal.pone.0232350 PMC7190152

[CR155] Wong, K. L. M., Restrepo-Méndez, M. C., Barros, A. J. D., & Victora, C. G. (2017). Socioeconomic inequalities in skilled birth attendance and child stunting in selected low and middle income countries: Wealth quintiles or deciles? *PLoS ONE*. 10.1371/journal.pone.0174823 28467411 10.1371/journal.pone.0174823 PMC5414946

[CR156] Yadav, A. K., & Jena, P. K. (2020). Maternal health outcomes of socially marginalized groups in India. *International Journal of Health Care Quality Assurance*. 10.1108/ijhcqa-08-2018-0212 32129579 10.1108/IJHCQA-08-2018-0212

[CR157] Yaya, S., Zegeye, B., Idriss-Wheeler, D., & Shibre, G. (2020). Inequalities in caesarean section in Burundi: Evidence from the Burundi Demographic and Health Surveys (2010–2016). *BMC Health Services Research*. 10.1186/s12913-020-05516-8 32664936 10.1186/s12913-020-05516-8 PMC7359587

[CR158] Yoseph, M., Abebe, S. M., Mekonnen, F. A., Sisay, M., & Gonete, K. A. (2020). Institutional delivery services utilization and its determinant factors among women who gave birth in the past 24 months in Southwest Ethiopia. *BMC Health Services Research,**20*(1), 265–265. 10.1186/s12913-020-05121-9 32228558 10.1186/s12913-020-05121-9 PMC7106731

[CR159] Yuan, B., Målqvist, M., Trygg, N., Qian, X., Ng, N., & Thomsen, S. (2014). What interventions are effective on reducing inequalities in maternal and child health in low- and middle-income settings? *A Systematic Review. BMC Public Health,**14*(1), 634–634. 10.1186/1471-2458-14-634 24952656 10.1186/1471-2458-14-634 PMC4083351

[CR160] Zahroh, R. I., Disney, G., Betrán, A. P., & Bohren, M. A. (2020). Trends and sociodemographic inequalities in the use of caesarean section in Indonesia, 1987–2017. *BMJ Global Health*. 10.1136/bmjgh-2020-003844 33380412 10.1136/bmjgh-2020-003844 PMC7780721

[CR161] Zegeye, B., Ahinkorah, B. O., Ameyaw, E. K., Budu, E., Seidu, A.-A., Olorunsaiye, C. Z., & Yaya, S. (2022). Disparities in use of skilled birth attendants and neonatal mortality rate in Guinea over two decades. *BMC Pregnancy and Childbirth*. 10.1186/s12884-021-04370-8 35062893 10.1186/s12884-021-04370-8 PMC8783403

[CR162] Zhao, P., Han, X., You, L., Zhao, Y., Yang, L., & Liu, Y. (2020). Maternal health services utilization and maternal mortality in China: A longitudinal study from 2009 to 2016. *BMC Pregnancy and Childbirth,**20*(1), 220–220. 10.1186/s12884-020-02900-4 32295528 10.1186/s12884-020-02900-4 PMC7161293

[CR163] La Barbera, S. (2023). Investing in Maternal Health: Economic Benefits and Policy Implications.

[CR164] Kurniasih, N.I.D., & Sulistyaningsih, I. (2019). Effort For Utilization Antenatal Care (ANC) In Pregnant Women: systematic literature review. *Proceeding International Conference*. https://prosiding.respati.ac.id/index.php/PIC/article/view/67

[CR165] Lawn, J., & Kerber, K. (2016). Opportunities for Africa’s newborns: practical data policy and programmatic support for newborn care in Africa; 2006. *Partnership for Maternal, Newborn and Child Health, Cape Town*. https://resourcecentre.savethechildren.net/document/opportunities-african-newborns-practical-data-policy-and-programmatic-support-newborn-care

[CR166] Majebi, N. L., Adelodun, M. O., & Chinyere, E. (2024). Maternal mortality and healthcare disparities: Addressing systemic inequities in underserved communities. *International Journal of Engineering Inventions*, *13*(9), 375–385. https://chwcentral.org/wp-content/uploads/Maternal-Mortality-and-Healthcare-Disparities-Addressing-Systemic-Inequities-in-Underserved-Communities.pdf

[CR167] Masselos, J. (2021). *Why is Maternal Health Crucial to Global Health and Development? HITLAB*. https://www.hitlab.org/post/why-is-maternal-health-crucial-to-global-health-and-development.

[CR168] Prevention, C. f. D. C. a. (2022). *Four in 5 pregnancy-related deaths in the U.S. are preventable. Centers for Disease Control and Prevention Online Newsroom*. https://archive.cdc.gov/www_cdc_gov/media/releases/2022/p0919-pregnancy-related-deaths.html

[CR169] Restrepo-Méndez, M. C., Barros, A. J. D., Requejo, J., Durán, P., Serpa, L. A. d. F., França, G. V. A., Wehrmeister, F. C., & Victora, C. G. (2015). Progress in reducing inequalities in reproductive, maternal, newborn,' and child health in Latin America and the Caribbean: an unfinished agenda. *Revista Panamericana de Salud Pública*, *38*(1). https://www.scielosp.org/pdf/rpsp/2015.v38n1/09-16/en 26506316

[CR170] Saldanha, I. J., Adam, G. P., Kanaan, G., Zahradnik, M. L., Steele, D. W., Danilack, V. A., Peahl, A. F., Chen, K. K., Stuebe, A. M., & Balk, E. M. (2023). Postpartum Care up to 1 Year After Pregnancy: A Systematic Review and Meta-Analysis. In: Agency for Healthcare Research and Quality (US), Rockville (MD). https://europepmc.org/article/NBK/nbk592637 37315166

[CR172] Vale, A. (2023). *80% of pregnancy-related deaths are preventable — here’s a closer look at maternal mortality in the US. Northwell Health*. https://www.northwell.edu/news/the-latest/80-of-pregnancy-related-deaths-are-preventable-a-look-at-maternal-mortality-in-us

[CR173] Waeni, J. (2023). *Molecular Signatures of Severe Acute Infections in Hospitalised African Children.* Open University (United Kingdom) ProQuest Dissertations & Theses. 10.21954/ou.ro.00016d38

[CR174] WB (2023). *World Bank Country and Lending Groups 2023.* https://datahelpdesk.worldbank.org/knowledgebase/articles/906519-world-bank-country-and-lending-groups

[CR175] WHO (2013). *Handbook on health inequality monitoring: with a special focus on low- and middle-income countries*. World Health Organization. https://go.exlibris.link/BDXTkm9c

[CR176] WHO (2016). *WHO recommendations on antenatal care for a positive pregnancy experience*. World Health Organization. https://www.who.int/publications/i/item/9789241549912 28079998

[CR177] WHO (2019). *Trends in maternal mortality 2000 to 2017: estimates by WHO, UNICEF, UNFPA, World Bank Group and the United Nations Population Division. World Health Organization*. https://iris.who.int/handle/10665/327596.

[CR178] WHO (2020). *Maternal mortality. World Health Organization*. https://www.who.int/news-room/fact-sheets/detail/maternal-mortality.

[CR179] WHO (2021). *Health Equity Assessment Toolkit (HEAT): Software for exploring and comparing health inequalities in countries*. *Built-in database edition. Version 4.0. Geneva, World Health Organization.* 10.1186/s12874-016-0229-9 PMC5069829 27760520

