# Supplementary material for: Inequality in Utilization of Maternal Healthcare Services in Low‑ and Middle‑Income Countries: A Scoping Review of the Literature
Source: Matern Child Health J. 2025 Jun 3;29(6):741–66. doi: 10.1007/s10995-025-04111-9 (PMC12206214; doi:10.1007/s10995-025-04111-9)
Supplement: Supplementary file 1 — Supplementary file1 (DOCX 19 KB) [file 10995_2025_4111_MOESM1_ESM.docx]

**PubMed Search String**

(("Prenatal Care"[mh] OR "Prenatal Care"[tw] OR "Delivery, Obstetric"[mh] OR "Obstetric care"[tw] OR "Maternity care"[tw] OR "Antenatal care"[tw] OR "Antenatal care services"[tw] OR "Postnatal Care"[mh] OR "Postnatal Care"[tw] OR "Postpartum Care"[tw] OR "Perinatal Care"[mh] OR "Perinatal Care"[tw] OR "Skilled birth delivery"[tw] OR "Institutional delivery"[tw] OR Skilled birth attendan*[tw] OR Healthcare deliver*[tw] OR "Health Care delivery"[tw] OR "Delivery care"[tw] OR "Pregnancy care"[tw] OR Facility based deliver*[tw] OR "Facility delivery"[tw] OR "Maternal Health"[mh] OR "Maternal Health"[tw] OR "Maternal Health Services"[mh] OR "Maternal Health Services"[tw] OR "Maternal care services"[tw] OR "Maternal Health Care"[tw] OR "Maternal Healthcare"[tw] OR "Maternal Healthcare Services"[tw] OR "Maternal Health Care Utilization"[tw] OR "Maternal Health Care Access"[tw]) AND ("Diversity, Equity, Inclusion"[mh] OR Equity[tw] OR Disparit*[tw] OR Equality[tw] OR Inequity[tw] OR Inequality[tw] OR "horizontal equity"[tw] OR discrepanc*[tw] OR "Unequal distribution"[tw] OR Discrimination[tw] OR Socioeconomic disparit*[tw] OR Socioeconomic inequalit*[tw] OR "Health Equity"[mh] OR "Health Equity"[tw] OR "Health inequities"[tw] OR Health Care Inequit*[tw] OR Health Care Inequalit*[tw] OR "Healthcare Disparities"[mh] OR "Healthcare Disparities"[tw] OR "Financial inequality"[tw] OR "Income inequity"[tw] OR "Income inequality"[tw] OR "Educational disparities"[tw] OR "Racial and ethnic disparities"[tw] OR Inequality measure*[tw] OR Ethnic disparit*[tw] OR "Geographical inequity"[tw] OR "Socioeconomic Disparities in Health"[mh] OR "Socioeconomic Disparities in Health"[tw]) AND ("Developing Countries"[mh] OR "Low-income countries"[tw] OR LMICs[tw] OR "Lower-middle-income countries"[tw] OR "Low- and middle-income countries"[tw] OR Afghanistan[tw] OR "Burkina Faso"[tw] OR Burundi[tw] OR "Central African Republic"[tw] OR "Central Africa"[tw] OR Chad[tw] OR Congo[tw] OR Eritrea[tw] OR Ethiopia[tw] OR Gambia[tw] OR Guinea[tw] OR Guinea-Bissau[tw] OR "North Korea"[tw] OR Liberia[tw] OR Madagascar[tw] OR Malawi[tw] OR Mali[tw] OR Mozambique[tw] OR Niger[tw] OR Rwanda[tw] OR "Sierra Leone"[tw] OR Somalia[tw] OR "South Sudan"[tw] OR Sudan[tw] OR Syria[tw] OR "Syrian Arab Republic"[tw] OR Togo[tw] OR Uganda[tw] OR Yemen[tw] OR Algeria[tw] OR Angola[tw] OR Bangladesh[tw] OR Belize[tw] OR Benin[tw] OR Bhutan[tw] OR Bolivia[tw] OR "Cabo Verde"[tw] OR Cambodia[tw] OR Cameroon[tw] OR Comoros[tw] OR "Côte d'Ivoire"[tw] OR Djibouti[tw] OR Egypt[tw] OR "El Salvador"[tw] OR Eswatini[tw] OR Ghana[tw] OR Haiti[tw] OR Honduras[tw] OR India[tw] OR Indonesia[tw] OR Iran[tw] OR Kenya[tw] OR Kiribati[tw] OR "Kyrgyz Republic"[tw] OR "Lao PDR"[tw] OR Lesotho[tw] OR Timor-Leste[tw] OR Mauritania[tw] OR Micronesia[tw] OR Mongolia[tw] OR Morocco[tw] OR Myanmar[tw] OR Nepal[tw] OR Nicaragua[tw] OR Nigeria[tw] OR Pakistan[tw] OR "Papua New Guinea"[tw] OR Philippines[tw] OR Samoa[tw] OR "São Tomé and Principe"[tw] OR Senegal[tw] OR "Solomon Islands"[tw] OR "Sri Lanka"[tw] OR Tajikistan[tw] OR Tanzania[tw] OR Tunisia[tw] OR Ukraine[tw] OR Uzbekistan[tw] OR Vanuatu[tw] OR Vietnam[tw] OR "West Bank and Gaza"[tw] OR Zambia[tw] OR Zimbabwe[tw] OR Albania[tw] OR "American Samoa"[tw] OR Argentina[tw] OR Armenia[tw] OR Azerbaijan[tw] OR Belarus[tw] OR "Bosnia and Herzegovina"[tw] OR Botswana[tw] OR Brazil[tw] OR Bulgaria[tw] OR China[tw] OR Colombia[tw] OR "Costa Rica"[tw] OR Cuba[tw] OR Dominica[tw] OR "Dominican Republic"[tw] OR Ecuador[tw] OR "Equatorial Guinea"[tw] OR Fiji[tw] OR Gabon[tw] OR Georgia[tw] OR Grenada[tw] OR Guatemala[tw] OR Guyana[tw] OR Iraq[tw] OR Jamaica[tw] OR Jordan[tw] OR Kazakhstan[tw] OR Kosovo[tw] OR "St. Lucia"[tw] OR Lebanon[tw] OR Libya[tw] OR Malaysia[tw] OR Maldives[tw] OR "Marshall Islands"[tw] OR Mauritius[tw] OR Mexico[tw] OR Moldova[tw] OR Montenegro[tw] OR Namibia[tw] OR "North Macedonia"[tw] OR Panama[tw] OR Paraguay[tw] OR Peru[tw] OR Romania[tw] OR "Russian Federation"[tw] OR Serbia[tw] OR "South Africa"[tw] OR "St. Vincent and the Grenadines"[tw] OR Suriname[tw] OR Thailand[tw] OR Tonga[tw] OR Turkey[tw] OR Turkmenistan[tw] OR Tuvalu[tw])) NOT ("Systematic Review"[pt] OR "Books and Documents"[pt] OR "Newspaper Article"[pt] OR Editorial[pt] OR Preprint[pt] OR News[pt] OR "Case Reports"[pt] OR Comment[pt] OR "Video-Audio Media"[pt] OR Review[pt] OR "Clinical Trial"[pt]) AND (2015:2023[pdat] AND English[la])

Filter: MEDLINE

**Description of Health inequality measures**

1. **CI – Concentration Index**

**Meaning:** A measure of income-related inequality in a health variable.
**Lay Explanation:** It shows whether a health outcome (like access to care or disease prevalence) is more common among the rich or the poor.

- **Value range:** -1 to +1.
  - **Negative:** More common among the poor.
  - **Positive:** More common among the rich.
  - **Zero:** Equal across income groups.

1. **CC – Concentration Curve**

**Meaning:** A graphical representation of the Concentration Index.
**Lay Explanation:** It’s a graph that shows how health outcomes are distributed across income levels.

- If the curve lies **below** the equality line → health is concentrated among the rich.
- If it’s **above** the line → health is concentrated among the poor.

1. **SII – Slope Index of Inequality**

**Meaning:** A regression-based measure of absolute inequality.
**Lay Explanation:** It tells you how much health differs between the richest and poorest, in terms of units (e.g., life expectancy years or disease rates).

- **Interpretation:** A higher value means a bigger gap between the rich and poor.

1. **RII – Relative Index of Inequality**

**Meaning:** A measure of **relative** health inequality.
**Lay Explanation:** It shows how many times better (or worse) health is for the richest compared to the poorest.

- **For example,** an RII of 2 = richest group has twice the health outcome as the poorest.

1. **PAF – Population Attributable Fraction**

**Meaning:** The proportion of cases that could be avoided if a risk factor were eliminated.
**Lay Explanation:** It answers: *“How much disease in the population is due to X (e.g., smoking)?”*

- **E.g.** If PAF for smoking and lung cancer is 70%, then 70% of lung cancer cases could be avoided without smoking.

1. **PAR – Population Attributable Risk**

**Meaning:** The absolute difference in disease rates with and without the risk factor.
**Lay Explanation:** It tells us how much **extra disease** exists in the population because of the risk factor.

- Unlike PAF (a percentage), PAR is an absolute number (e.g., 15 cases per 1,000 people).

1. **BGV – Between-Group Variance**

**Meaning:** A measure of health inequality between defined groups (e.g., income quintiles).
**Lay Explanation:** It tells you how much of the overall health difference is because of differences **between** groups (not within them).

- Higher BGV means more inequality between, say, poor and rich.

1. **CV – Coefficient of Variation**

**Meaning:** A normalized measure of variability.
**Lay Explanation:** It tells you how **spread out** a health variable is relative to the average.

- **For example,** a high CV in access to care means some people get much more care than others.

In health economics, **Blinder-Oaxaca** and **Fairlie** decomposition methods are statistical techniques used to understand differences in outcomes (like income, health status, or healthcare access) between two groups (e.g., men vs. women, insured vs. uninsured, ethnic groups).

1. **Blinder-Oaxaca Decomposition**

**Formal definition**:
Originally developed in labor economics (Blinder, 1973; Oaxaca, 1973), this method decomposes the **mean difference in an outcome** between two groups into:

- A part **explained by differences in characteristics** (e.g., education, age, income), and
- A part **unexplained**, which may reflect discrimination, omitted variables, or differences in returns to characteristics.

**Use in health economics**:
Used to analyze health inequalities. For example, it can show how much of the difference in access to care between urban and rural populations is due to observable factors like income, education, and insurance status.

**Layperson explanation**:
It answers: *“Why do two groups have different average outcomes?”*
It separates the difference into:

- The part due to having different resources or characteristics.
- The part that can't be easily explained—possibly due to bias or unmeasured factors.

1. **Fairlie Decomposition**

**Formal definition**:
An extension of Blinder-Oaxaca for **non-linear models**, especially **binary outcomes** (e.g., whether someone has access to healthcare or not). Developed by Fairlie (2005), it allows decomposing differences in outcomes when the dependent variable is not continuous.

**Use in health economics**:
Useful for studying disparities in yes/no outcomes, such as immunization uptake, insurance coverage, or disease incidence between population groups.

**Layperson explanation**:
It answers: *“Why do more people in one group have a certain outcome compared to another group?”*
It breaks down the gap into:

- How much is due to different traits (like income or education).
- How much is due to other, harder-to-explain reasons.
