# Supplementary material for: Inequality in Utilization of Maternal Healthcare Services in Low‑ and Middle‑Income Countries: A Scoping Review of the Literature
Source: Matern Child Health J. 2025 Jun 3;29(6):741–66. doi: 10.1007/s10995-025-04111-9 (PMC12206214; doi:10.1007/s10995-025-04111-9)
Supplement: Supplementary file 2 — Supplementary file2 (DOCX 51 KB) [file 10995_2025_4111_MOESM2_ESM.docx]

**Supplementary Table 1: Summary analysis of the studies of inequality in utilization of MHS in LIC, LwMIC, UMIC and LMIC**

| **Study** | **Location** | **Study Design** | **Inequality Measures** | **Outcome measure (Utilization of)** | **Covariate** |
| --- | --- | --- | --- | --- | --- |
| **Single-country studies: low- income counties (n = 19)** | | | | | |
| Shibre et al., 2023 | Ethiopia | Demographic and Health Surveys (2000, 2005, 2011, and 2016) | CC, and Decomposition of CI | Early ANC, 4+ ANC visits, quality ANC, PNC within 2 days (women) | Women decision-making, social independence, attitude toward violence, age at pregnancy, residence, region, mother’s education, partner’s education, mother’s religion, media exposure, wealth status, mother’s occupation, partner’s occupation, survey timing, and ethnicity |
| Tsegaye et al., 2022 | Ethiopia | Demographic and Health Surveys (2000, 2005, 2011, 2016 and 2019) | CC, CI, Ratio, and multivariate decomposition regression analysis | 1+ ANC visits, and 4+ ANC visits | Wealth status, women's educational status |
| Tarekegn et al., 2022 | Ethiopia | Demographic and Health Survey (2000, 2005, 2011, 2016, and 2019) | CI, and Ratio | SBA | Wealth status, education, residence |
| Kakama et al., 2022 | Uganda | Demographic and Health Surveys 2006, 2011, and 2016 | Ratio, and CI | ANC by SP, SBA and FBD | Wealth status, and residence |
| Tsawe & Susuman, 2022 | Sierra Leone | Demographic and Health Surveys 2008, 2013, and 2019 | CC, CI, and Ratio | 4+ ANC visits, ANC by SP, SBA, and FBD | Wealth status, education, and residence |
| Yaya et al., 2020 | Burundi | Demographic and Health Surveys of 2010, and 2016 | Difference, PAR and SII | C-section delivery | Wealth status, education, residence, and region |
| Atake, 2020 | Togo | Togo Demographic and Health Survey 1998, and 2013 | CC, CI, and Decomposition of CI | 5+ ANC visits, and FBD | Wealth status, age, women's education, residence, religion, health insurance, and husband's education |
| Daka et al., 2020 | Ethiopia | Demographic and Health Surveys 2016 | CC, CI, and Ratio | ANC visits by SP, 4+ ANC visits, FBD, and PNC within 2 days (Newborn) | Wealth status |
| Shibre et al., 2020 | Ethiopia | Demographic Health Surveys (2000, 2005, 2011 and 2016) | Ratio, PAR, SII, and CI | C-section delivery | Wealth status, education, residence, and region |
| Jalloh et al., 2019 | Sierra Leone | Demographic and Health Surveys 2008, and 2013 | CI, and CC | 5+ ANC visits, and 4+ PNC visits | Wealth status |
| Gebre et al., 2018 | Ethiopia | Ethiopia Demographic and Health Surveys (2000 and 2016) | CC, CI, and Decomposition of CI | 4+ ANC visits, SBA and PNC within 2 days (women) | Wealth status, age, birth order, residence, occupation, education, marital status, media exposure |
| Mezmur et al., 2017 | Ethiopia | Demographic and Health Surveys (2000, 2005 and 2011) | CC, CI, and Decomposition of CI | 1+ ANC visit, 4+ ANC visits, ANC in first trimester, FBD, and SBA | Wealth status, age, birth order, maternal education, occupation, ethnic origin, religious affiliation, and media access |
| Tesfaye et al., 2017 | Ethiopia | Demographic and Health Survey (2000, 2005, 2011 and 2016) | Difference, CC, and CI | SBA | Wealth status, education |
| Ambel et al., 2017 | Ethiopia | Demographic and Health Surveys (2000, 2005, 2011, and 2014) | CC, CI, and Decomposition of CI | 4+ ANC visits, and SBA | Wealth status, age, education, residence, and region |
| Bobo et al., 2017 | Ethiopia | Demographic and Health Survey 2014 | CC, CI, and Ratio | 1+ ANC visits by SP, SBA, FBD, and PNC within 2 days (women) | Wealth status |
| Kim et al., 2016 | Afghanistan | Afghanistan Mortality Survey 2010 | CI, CC, and Decomposition of CI | Any ANC, and FBD | Wealth status, Age, education, and residence |
| Akseer et al., 2016 | Afghanistan | Multiple Indicator Cluster Survey 2010-2011 | Difference, Ratio, CI, SII, and Equiplots | 4+ ANC visits, ANC by SP, and SBA | Wealth status |
| Memirie et al., 2016 | Ethiopia | Demographic and Health Survey 2005, and 2011 | Horizontal inequity, and Decomposition of CI | SBA | Wealth status, educational attainment of household head, educational attainment of partner, and residence |
| Onarheim et al., 2015 | Ethiopia | Demographic and Health Surveys 2011 | CI, and Decomposition of CI | 4+ ANC visits, and SBA | Wealth status, age, education, household head, previous healthcare use, religion, residence, and region |
| **Single-country studies: lower middle-income counties (n = 70)** | | | | | |
| Shreezal & Adhikari, 2023 | Nepal | Demographic Health Survey 2011, and 2016 | CC, Gini index, Decomposition of CI, and Oaxaca-blinder decomposition analysis | 4+ ANC visits, PNC within 2 months (newborn), and SBA | Wealth status, age, sex of household head, marital status, religion, number of children, residence, ecological zones, mother’s education, partner’s education, and distance to health facility |
| Tetteh et al., 2023 | Ghana | Demographic and Health Survey 1993, 1998, 2003, 2008, and 2014 | Ratio, Difference, PAF and PAR | SBA | Wealth status, education, residence, and region |
| Chowdhury et al., 2023 | Bangladesh | Demographic and Health Surveys 2004, 2007, 2011, 2014, and 2017/18 | Difference, Ratio, PAF, and PAR | SBA | Wealth status, education, residence, and region |
| Ali et al., 2023 | Nepal | Demographic and Heath Survey 1996, 2001, 2006, 2011, and 2016 | SII, RII, and Fairlie decomposition analysis | 4+ ANC visits, FBD, and 1+ PNC visits | Wealth status, age, education, parity, time to reach health center, caste, and partner’s education |
| Chauhan & Radkar, 2023 | India | National Family Health Survey 1992/93, 1998/1999, 2005/06, 2015/16, and 2019/21 | CC, CI, and Decomposition of CI | C-section delivery | Wealth status, age, caste, religion, education, residence, region, media exposure, children ever born, BMI, health insurance, child size at birth, and multiple births |
| Rahman et al., 2022 | Bangladesh | Demographic and Health Survey 2017/18 | CC, and Decomposition of CI | FBD | Wealth status, residence, region, age, education, age at first birth, BMI, mother’s empowerment, employment, no. of ANC visits, husband’s education, and husband’s occupation |
| Kim et al., 2022 | Cambodia | Demographic and Health Surveys 2005, 2010, and 2014 | Ratio, and Equiplots | Quality ANC, and PNC before discharge | Wealth status, education, and residence |
| Seidu et al., 2022 | Ghana | Demographic and Health Survey 1998, 2003, 2008, and 2014 | PAF, PAR, Ratio, and Difference | 4+ ANC visits | Wealth status, education, residence, and region |
| Rahman et al., 2022 | Bangladesh | Demographic and Health Survey 2007, and 2017/2018 | CC, and Decomposition of CI | FBD | Wealth status, age, education, residence, birth order, region, BMI, employment, ANC visits, partner’s education, partner’s occupation, sanitation facilities, and improved water |
| Okyere et al., 2022 | Ghana | Demographic and Health Surveys 1998, 2003, 2008, and 2014 | Difference, Ratio, PAF, and PAR | C-section delivery | Wealth status, education, residence, and region |
| Taleb El Hassen et al., 2022 | Mauritania | Multiple indicator cluster surveys 2007, and 2015 | CC, CI, and Decomposition of CI | SBA | Wealth status, age, birth order, residence, region, and education |
| Lukwa et al., 2022 | Zimbabwe | Demographic Health Survey 2015 | CC, CI, and Decomposition of CI | 4+ ANC, SBA, and PNC within 2 days (women) | Wealth status, age, employment, education, partner’s education, residence, sex of household head, birth order, distance to facility, and media access |
| Shirisha et al., 2022 | India | National Family Health Survey 2005/06, and 2015/16 | SII, CI, and Equiplots | 4+ ANC visits, FBD, PNC within 2 days (Newborn), and PNC within 2 days (women) | Wealth status |
| Zegeye et al., 2022 | Guinea | Demographic and Health Surveys 1999, 2005, and 2012; and Multiple Indicator Cluster Survey 2016 | Difference, Ratio, PAF, and PAR | SBA | Wealth status, education, residence, and region |
| Sk et al., 2022 | India | National Family Health Survey 2005/06, and 2015/16 | CC, CI, and Decomposition of CI | Full ANC, SBA, and PNC within 2 days | Wealth status, residence, age in years, birth order, education, caste, and mass media exposure |
| Fagbamigbe & Oyedele, 2022 | Nigeria | Demographic and Health Surveys 1990, 2003, 2008, 2013, and 2018 | Multivariate decomposition analysis | SBA | Age, education, partner’s education, employment, sex of household head, media access, wealth status, no. of ANC visits, residence, religion, distance to health facility, ethnicity, family mobility, wanted last child, and birth interval |
| Godha & Hotchkiss, 2022 | India | Demographic and Health Surveys 2005/06, and 2015/16 | CI | 3+ ANC visits, FBD, and PNC within 2 days (women) | Residence, wealth status, and states performance |
| Gandhi et al., 2022 | India | National Family Health Survey 2005/06, and 2015/16 | CI | Any ANC, SBA, and any PNC | Wealth status |
| Anarwat et al., 2021 | Ghana | Multiple Indicator Cluster Survey 2011 | CI | ANC by SP, SBA, and FBD | Wealth status, education, and residence |
| Ekholuenetale et al., 2021 | Ghana | Malaria Indicator Survey 2019 | CI | 8+ ANC visits | Wealth status, and education |
| Gandhi et al., 2021 | India | National Family Health Survey 2015/16 and Public Affairs Index 2016 | Equiplot | 4+ ANC visits and SBA | Wealth status |
| Bintabara, 2021 | Tanzania | Demographic Health Surveys 2004, 2010, and 2016 | CC, CI, and Decomposition of CI | SBA, and FBD | Wealth status, age, education, marital status, employment, and region |
| Sapkota et al., 2021 | Nepal | Demographic and Health Survey 2001, 2006, 2011, and 2016 | CC, and CI | 4+ ANC visits, FBD, and C-section delivery | Wealth status, and region |
| Nguyen et al., 2021 | Vietnam | Multiple Indicator Cluster Surveys 2000, 2006, 2011, 2013/2014; and Demographic and Health Surveys 2002 | SII, RII, and CI | 1+ ANC visits, 4+ ANC visits, SBA, and FBD | Wealth status, Residence, and Region |
| Mishra et al., 2021 | India | National Family Health Survey 2015/2016 | CC, CI, and Decomposition of CI | 4+ ANC visits, FBD, and PNC within 2 weeks (women) | Wealth status, age, education, birth order, religion, residence, media exposure, and sex of household head |
| Bintabara & Basinda, 2021 | Tanzania | Demographic and Health Survey 2004, 2010, and 2016 | CC, CI, and Decomposition of CI | 4+ ANC visits, and quality ANC | Wealth status, age, residence, education, marital status, and employment |
| Paul, 2021 | India | National Family Health Survey 2005/06, and 2015/16 | Ratio, Difference, CC, CI, and SII | ANC in first trimester, 4+ ANC visits, FBD, SBA, and PNC within 2 days/ 48h (women) | Wealth status |
| Shibre et al., 2021 | Mauritania | Mauritania Multiple Indicator Cluster Surveys 2011, and 2015 | Difference, Ratio, PAR, and PAF | 4+ ANC visits | Wealth status, education, residence, and region |
| Kpodotsi et al., 2021 | Ghana | Demographic and Health Survey 2014 | CC, CI, and Decomposition of CI | SBA | Wealth status, age at birth, marital status, employment, residence, region, education, partner’s education, sex of household head, health insurance, and mother’s autonomy |
| Ali et al., 2021 | India | National Family Health survey 2015/16 | CC, CI, and Decomposition of CI | Full ANC, SBA, and PNC within 48h (women) | Region, wealth status, age, birth order, sex of child, sex of household head, education, autonomy, caste, media exposure, and religion |
| Bhusal, 2021 | Nepal | Multiple Indicator Cluster Survey 2019 | CC, and Decomposition of CI | FBD | Wealth status, age, ANC visits, education, and residence |
| Chauhan & Jungari, 2021 | India | National Family Health Survey 2015/2016 | CC, and CI | Full ANC, FBD, and PNC within 2 days (women) | Wealth status, and region |
| Pandey et al., 2021 | Nepal | Demographic and Health Survey 2001, 2006, 2011, and 2016 | Ratio, Difference, CI, PAR, and SII | 4+ ANC visits, and SBA | Wealth status, and residence |
| Ali et al., 2020 | India | National Family Health Survey 2005/06, and 2015/16 | CC, and Decomposition of CI | Full ANC, SBA, and PNC within 2 days (women) | Wealth status, residence, age, birth order, education, religion, and caste |
| Shibre et al., 2020 | Angola | Demographic and Health Survey 2015 | Ratio, Difference, PAF and PAR | 4+ ANC visits | Wealth status, education, residence, and region |
| Khadr, 2020 | Egypt | Demographic and Health Survey 1995, and 2014 | CI, and Decomposition of CI | 4+ ANC visits | Wealth status, age, education, region, husband’s education, toilet type, birth attributes, experience of terminated pregnancy, and previous birth experience |
| Pulok et al., 2020 | Bangladesh | Demographic and Health Survey 2011, and 2014 | CI, and Decomposition of CI | FBD, SBA, and C-section delivery | Wealth status, age, age at marriage, education, pregnancy complications, religion, parity, 4+ ANC visits, media exposure, microcredit involvement, husband’s education, residence, and region |
| Thapa et al., 2020 | Nepal | Demographic Health Surveys 2001, 2006, 2011, and 2016 | Ratio, Difference, and Equiplot | ANC by SP, and SBA | Wealth status, and education |
| Panda et al., 2020 | India | National Family Health Survey 2005/06, and 2015/2016 | CC, CI, and Decomposition of CI | C-section delivery | Wealth status, age, residence, region, education, religion, caste, birth order, ANC, place of birth, and pregnancy complications |
| Panda et al., 2020 | India | National Family Health Survey 2015/2016 | Ratio, and Difference | 1+ ANC visits by SP, and SBA | Wealth status, and residence |
| Shibre et al., 2020 | Tanzania | Demographic and Health Surveys 1996, 1999, 2004, 2010, and 2015 | Ratio, Difference, SII, and RII | C-section delivery | Wealth status, education, residence, and region |
| Okoli et al., 2020 | Nigeria | Demographic Health Surveys 2003, 2008, 2013, and 2018 | Ratio, Difference, CI, Theil index, and BGV | 4+ ANC visits by SP, FBD, and SBA | Wealth status, education, residence, and geopolitical zone |
| Krishnamoorthy et al., 2020 | India | National Family Health Survey 2015/2016 | CC, CI, and Theil index | 4+ ANC visits, FBD, PNC within 24h (women/newborn), and PNC within 48h (women/ newborn) | Wealth status, residence, and region |
| Novignon et al., 2019 | Ghana | Demographic and Health Surveys 2003, 2008, and 2014 | CC, CI, and Decomposition of CI | 4+ ANC visits, and SBA | Wealth status, education, residence, age, health insurance, and region |
| Ushie et al., 2019 | Nigeria | Demographic and Health Survey 2013 | CC, and CI | C-section delivery | Wealth status |
| Kien et al., 2019 | Vietnam | Multiple Indicator Cluster Survey 2006, 2011, and 2014 | CI | Quality ANC | Wealth status |
| Amporfu & Grépin, 2019 | Ghana | Demographic and Health Survey 2003, 2008, and 2014 | Oaxaca/ Blinder/ Reimers/ Cotton decomposition analysis | FBD | Wealth status, residence, education, age, parity, antenatal care visits, ethnicity, religion, region, marital status, distance from a health facility, and pregnancy complications |
| Lam et al., 2019 | Vietnam | Multiple Indicator Cluster Survey 2014 | CC, and CI | PNC within 2 days (newborn) | Wealth status |
| Kumar et al., 2019 | India | National Family Health Survey 2015/2016 | CC, and CI | Full ANC | Residence, education, caste, and region |
| Guilmoto & Dumont, 2019 | India | National Family and Health Survey 2015/2016 | CC, and Gini coefficient | C-section delivery | Wealth status |
| Yadav et al., 2019 | India | National Sample Survey Office unit-level data 1995, 2004, and 2014 | Blinder-Oaxaca decomposition analysis | Any ANC, FBD, and any PNC | Scheduled tribes, scheduled castes, wealth status, age, education, residence, water facilities, toilet facilities, and region |
| Dankwah et al., 2019 | Ghana | Demographic and Health Survey 2014 | CC, and CI | C-section delivery | Wealth status |
| Nwosu & Ataguba, 2019 | Nigeria | Demographic and Health Survey 2013 | CC, CI, and Decomposition of CI | 4+ ANC visits | Wealth status, region, residence, education, age, spousal education, women’s autonomy, health worker attitude, religion, sex of household head, and distance to facility |
| Keats et al., 2018 | Kenya | Demographic and Heath Survey 2003, 2008, and 2014 | Ratio, Difference, CI, SII, and Equiplots | ANC by SP, 4+ ANC visits, and SBA | Wealth status |
| Abekah-Nkrumah, 2018 | Ghana | Demographic and Health Survey 1998, and 2014 | Oaxaca decomposition analysis | 4+ ANC visits, and SBA | Age, birth order, women and partner’s education, ethnicity, religion, residence, wealth status, and region |
| Khan et al., 2018 | Bangladesh | Demographic and Health Survey 2004, and 2014 | CC, CI, and Decomposition of CI | C-section delivery | Wealth status, age, residence, region, education, BMI, birth interval, children ever born, and no. of ANC visits |
| Pulok et al., 2018 | Bangladesh | Demographic and Health Survey, 2014 | CC, and CI | 1+ ANC visit, 4+ ANC visits, ANC by SP, FBD, SBA, and C-section delivery | Wealth status, residence, and region |
| Rahman et al., 2017 | Bangladesh | Demographic Health Surveys 2011, and 2014 | Ratio, Difference, and CI | 4+ ANC visits | Wealth status |
| Mehata et al., 2017 | Nepal | Family Health Survey 1996; and Demographic and Health Surveys 2001, 2006, and 2011 | Ratio, Difference, and CI | 4+ ANC visits, FBD, and C-section delivery | Wealth status |
| Bayati et al., 2017 | Iran | Multiple Indicators of Demographics and Health Survey 2011 | Gini coefficient | SBA | Women’s population |
| Adeyanju et al., 2017 | Nigeria | Demographic and Heath Survey 1990, and 2008 | CC, CI, and Decomposition of CI | ANC by SP, and SBA | Wealth status, age, region, religion, and education |
| Vellakkal et al., 2017 | India | District Level Household and Facility Surveys 1995/99, 2000/04, 2007/08, and 2011/12; and Annual Health Survey 2011/12 | RII | 3+ ANC visits, and FBD | Wealth status |
| Asamoah & Agardh, 2017 | Ghana | Demographic and Health Surveys 2003, 2008, and 2014 | PAF | 4+ ANC visits, and SBA | Wealth status, education, and residence |
| Himanshu & Källestå, 2017 | India | District Level Household and Facility Surveys 2006/07, and 2012/13 | Theil index | Any ANC, full ANC, and SBA | Inter-district inequality |
| Kamal et al., 2016 | Bangladesh | Maternal Mortality and Health Care Survey 2001, and 2010 | Ratio, and CI | ANC by SP, and FBD | Wealth status, and residence |
| Hodge et al., 2016 | Philippines | Demographic and Health Survey 2013 | Fairlie decomposition analysis | FBD | Wealth status, region, distance to facility, education, multiple birth, age, employment, watches TV, previously terminated pregnancy, religion, maternal status, partner’s education, and partner’s employment |
| Agho et al., 2016 | Nigeria | Demographic and Health Surveys 2013 | PAR | PNC within the first 6 weeks | Residence, wealth status, education, mothers’ knowledge of delivery-related complications, access to media, and perceived size of the baby at birth |
| Pulok et al., 2016 | Bangladesh | Demographic and Health Survey 2004, 2007, and 2011 | CC, CI, and Ratio | 4+ ANC visits, ANC by SP, FBD, SBA, and C-section delivery | Wealth status, and residence |
| Paredes, 2016 | Philippines | Demographic and Health Survey 2008, and 2013 | CC, CI, and Decomposition of CI | Full ANC, FBD, and C-section delivery | Age, education, women’s union status, wealth status, residence, health insurance, age and sex of household head, and family size |
| Anwar et al., 2015 | Bangladesh | Demographic and Health Surveys 1993/1994, 1996/1997, 1999/2000, 2004, 2007, and 2011 | Ratio, Difference, and CI | Any ANC, FBD, and C-section delivery | Wealth status |
| **Single-country studies: upper middle-income counties (n = 15)** | | | | | |
| Fonseca et al., 2022 | Brazil | Live Birth Information System 2014, and 2020 | Ratio, and Difference | 7+ ANC visits | Age, Schooling, and race/ skin color |
| Rios-Quituizaca et al., 2022 | Ecuador | Reproductive Health Survey 2004; and National Health and Nutrition Survey 2012 | Equiplots, and Mean difference from the best | 4+ ANC visits, SBA, and FBD | Ethnicity |
| Rios Quituizaca et al., 2021 | Ecuador | National health surveys 1994, 1999, 2004, and 2012 | Mean difference from best performing subgroup, and Theil index | 4+ ANC visits, and FBD | Wealth status, and residence |
| Hernández- Vásquez et al., 2021 | Peru | Demographic and Family Health Surveys 2009, and 2018 | CI, SII, RII and Equiplot | C-section delivery | Wealth status, education, residence, and region |
| Quizhpe et al., 2020 | Ecuador | Living Standards Measurement Surveys 2006, and 2014 | SII | SBA | Residence, ethnicity, education, and wealth status |
| Zahroh et al., 2020 | Indonesia | Demographic and Health Surveys 1991, 1994, 1997, 2002, 2007, 2012, and 2017 | Ratio, and Difference | C-section delivery | Wealth status, education, residence, place of birth, and region |
| Sanogo & Yaya, 2020 | Gabon | Demographic and Health Surveys 2012 | CC, and CI | 4+ ANC visits, FBD, and PNC within 6 weeks | Wealth status, and health insurance |
| Flores et al., 2019 | Brazil | National Health Survey 2013 | CI, and SII | Quality ANC | Wealth status |
| Silva et al., 2018 | Brazil | Demographic and Maternal and Child Health Survey 2006; and National Health Survey 2013 | Ratio, Difference, CI, SII, and Equiplots | 6+ ANC visits, and quality ANC | Wealth status, and residence |
| Mallmann et al., 2018 | Brazil | Live Births Information System data 2000-2015 | Difference | 7+ ANC visits | Maternal education, and race/ethnicity |
| Fan et al., 2017 | China | National Health Service Survey 2008, and 2013 | CI | 5+ ANC visits, and 3+ PNC visits within 6 weeks | Residence, and wealth status |
| Nababan et al., 2017 | Indonesia | Demographic and Health Surveys 1991, 1994, 1997, 2002/03, 2007, and 2012 | Ratio, Difference, and CI | Any ANC, 4+ ANC visits, FBD, and C-section delivery | Wealth status |
| Liang et al., 2017 | China | National Maternal and Child Health Annual Report office 2000‐2013; National Health Statistics Yearbook 2015; and China Women and Children Statistical Information 2014 | Theil index, and BGV | 1+ ANC visit, 5+ ANC visits, FBD, C-section delivery, and more than 1 PNC (newborn) | Residence, and region |
| Wabiri et al., 2016 | South Africa | National HIV Prevalence, Incidence, Behaviour and Communication Surveys 2008, and 2012 | SII, RII, and Equiplots | Any ANC, 4+ ANC visits, and SBA | Wealth status |
| França et al., 2016 | Brazil | Demographic Health Surveys 1986, 1996, and 2006; and National Health Survey 2013 | CI, SII, and Equiplot | 1+ ANC visits, 4+ ANC visits, 1st ANC in first trimester, FBD, and C-section delivery | Wealth status, and residence |
| **Multi-country studies (n = 28)** | | | | | |
| Misu & Alam, 2023 | Bangladesh, and Pakistan | Demographic and Health Survey 2017/2018 | CC, CI, Difference, Ratio, SII, and Equiplot | ANC visits in 1st trimester, 4+ ANC visits, SBA, and FBD | Age, education, residence, women as household head, household size, employment, wealth status, husband’s education, wanted last child, last live birth order, and pregnancy termination history |
| Misu & Alam, 2023 | Bangladesh, and Pakistan | Demographic and Health Survey 2017/2018 | CC, CI, Difference, Ratio, SII, and Equiplots | PNC within 2 days by SP (Newborn & women), and Adequate PNC (Newborn) | Age, education, residence, women as household head, household size, employment, wealth status, husband’s education, wanted last child, last live birth order, sex of last birth, pregnancy termination history, media exposure, women’s autonomy, facility delivery, mode of delivery, and no. of ANC visits |
| Ahinkorah et al., 2022 | 28 Sub-Saharan African countries | Demographic and Health Surveys: 2010-2020 | Multivariate decomposition regression analysis | C-section delivery | Residence, wealth status, parity, age, child size at birth, marital status, religion, health insurance, employment, education, ANC visits, sex of household head, sex of child, media exposure, and partner’s education |
| Leventhal et al., 2021 | 36 LMICs | Demographic and Health Surveys: 2011-2018; Multiple Indicator Cluster Surveys: 2014, 2015 | CI, and SII | 4+ ANC visits, FBD, PNC within 2 days (women), and PNC within 2 days (newborn) | Wealth status |
| Bobo et al., 2021 | Burundi, Ethiopia, Malawi, Rwanda, Uganda, Kenya, Tanzania, Zambia, and Zimbabwe | Demographic and Health Surveys: Burundi (2016), Ethiopia (2016), Malawi (2016), Rwanda (2013/14), Uganda (2016), Kenya (2014), Tanzania (2015), Zambia (2018), and Zimbabwe (2015) | CC, and CI | 4+ ANC visits, and quality ANC | Wealth status, and residence |
| Dewau et al., 2021 | Ethiopia, Uganda, Kenya, and Tanzania | Demographic and Health Surveys: Ethiopia (2016), Uganda (2016), Kenya (2014), and Tanzania (2015) | Oaxaca, Blinder, Reimers, Cotton decomposition analysis | FBD | Residence |
| Bobo et al., 2021 | 25 Sub-Saharan African countries | Demographic and Health Surveys: 2013-2018 | CI, and Equiplot | SBA, and C-section delivery | Residence, and countries |
| Selebano & Ataguba, 2021 | 12 Sub-Saharan African Countries | Demographic and Health Surveys: 2006-2016 | CI, and Decomposition of CI | 4+ ANC visits | Wealth status, age no. of children, region, residence, education, employment, and sex of household head |
| Anindya et al., 2021 | 39 LMICs | Demographic and Health Surveys: 2014-2018 | Ratio, Difference, CI, SII, and RII | Any ANC by SP, 4+ ANC, Quality ANC, SBA, PNC within 24h (Newborn), and PNC by SP | Wealth status |
| Ogundele et al., 2020 | Burkina Faso, Niger, Nigeria, Ghana, and Senegal | Demographic and Health Survey: Burkina Faso (2010), Niger (2012), Nigeria (2013), Ghana (2014) and Senegal (2016) | Fairlie decomposition technique | 4+ ANC visits, FBD, and C-section delivery | Wealth status, age, children ever born, education, residence, region, religion, distance to facility, marital status, and pregnancy complications |
| Wehrmeister et al., 2020 | 60 LMICs | Demographic and Health Surveys: 2010-2016; Multiple Indicator Cluster Surveys: 2010-2016 | CI, SII, and Equiplot | 1+ ANC visits, 4+ ANC visits, SBA, and PNC within 2 days/ 48h (newborn) | Wealth status |
| Abekah-Nkrumah, 2019 | 30 Sub-Saharan African countries | Demographic and Health Surveys: 1998-2016 | CI | 4+ ANC visits, 8+ ANC visits, FBD, and SBA | Wealth status |
| Lohela et al., 2019 | 72 LMICs | Demographic and Health Surveys: 1990-2016 | Equiplots | FBD | Wealth status, and education |
| Arsenaul et al., 2018 | 91 LMICs | Demographic and Health Surveys 2007-2016; and Multiple Indicator Cluster Surveys 2010-2016 | SII, and RII | Quality ANC, and 1+ ANC by SP | Country income group |
| Huda et al., 2018 | Bangladesh, Pakistan, and Nepal | Demographic and health surveys: Bangladesh (2014), Pakistan (2012/13), and Nepal (2010/11) | CC, CI, horizontal inequity, and decomposition of CI | FBD | Wealth status, pregnancy complications, pregnancy termination history, age, employment, education, husband’s education, residence, and region |
| Goli et al., 2018 | Ethiopia, Bangladesh, Nepal, and Zimbabwe | Demographic Health Surveys: Nepal (2011), Bangladesh (2011), Ethiopia (2011), Zimbabwe (2010/11) | CC, and Decomposition of CI | 4+ ANC visits, FBD, and PNC within 2 days | Wealth status, residence, education, employment, birth order, husband’s education, and risky age of childbearing |
| Ravit et al., 2018 | Benin, and Mali | Demographic and Health Surveys: Benin (2001, 2006, 2011/2012), and Mali (2001, 2006, 2012/13) | CC | FBD, and C-section delivery | Wealth status |
| Ogundele et al., 2018 | Ghana, and Nigeria | Demographic and Health Surveys: Ghana (2003, 2008, 2014), and Nigeria (2003, 2008, 2013) | CC, and CI | ANC in 1st trimester, SBA, and C-section delivery | Wealth status |
| De La Torre et al., 2018 | Brazil, and Colombia (UMIC) | Demographic Health Surveys: Brazil (2006), and Colombia (2010) | CI, and Decomposition of CI | 4+ ANC visits, SBA, C-section delivery, and PNC within 2 months after birth (women) | Wealth status, age, wanted pregnancy, no. of alive born children, marital status, race, education, health insurance, region, residence, and place of delivery |
| Rahman et al., 2017 | Afghanistan, Bangladesh, India, Nepal, and Pakistan | Demographic and Health Surveys: Bangladesh (2014), Afghanistan (2014); Living Condition Survey: Afghanistan (2015); Household Income and Expenditure Survey: Bangladesh (2010); National Sample Survey Organization: India (2014); District Level Household Survey: India (2012); Annual Health Survey: Nepal (2015); Multiple Indicator Cluster Surveys: Nepal (2014), Pakistan (2014); Social and Living Standards Measurement Survey: Pakistan (2014) | SII, RII, and Equiplot | 1+ ANC visits, 4+ ANC visits, SBA, and FBD | Wealth status |
| Wong et al., 2017 | 46 LMICs | Demographic and Health Surveys: 2003-2013 | Ratio, Difference, CI, SII, and Equiplots | SBA | Wealth status |
| Boatin et al., 2017 | 72 LMICs | Demographic and Health Surveys: 2000-2004, 2010-2014; Multiple Indicator Cluster Surveys: 2010-2014 | Ratio, Difference, Gini index, and Equiplot | C-section delivery | Wealth status |
| Hosseinpoor et al., 2016 | Bangladesh, Egypt, Ghana, and Zimbabwe | Demographic and Health Survey: Bangladesh (1996, 1999, 2004, 2007), Egypt (1995, 2000, 2005, 2008), Ghana (1998, 2003, 2008), and Zimbabwe (1999, 2005, 2010) | Ratio, Difference, PAR, PAF, CV, Index of dissimilarity, Mean difference from mean, BGV and Theil index | 1+ ANC visits, and SBA | Region |
| McKinnon et al., 2016 | 48 LMICs | Demographic and Health Surveys: 2006-2012 | SII, and RII | 4+ ANC visits, FBD, and C-section delivery | Region |
| Do et al., 2015 | Ghana, Rwanda, and Philippines | Demographic Health Surveys: Ghana (2008), Rwanda (2005), and Philippines (2008) | CI, and Decomposition of CI | FBD | Wealth status, education, parity, age, and religion |
| Alam et al., 2015 | Ethiopia, Madagascar, Uganda, Cameroon, Zambia, and Zimbabwe | Demographic and Health Surveys: Ethiopia (2000, 2005, 2011), Madagascar (1997, 2003/04, 2008/09), Uganda (2000/01, 2006, 2011), Cameroon (1998, 2004, 2011), Zambia (1996, 2001/02, 2007), and Zimbabwe (1999, 2005/06, 2010/11) | Ratio, Difference, and CI | 4+ ANC visits, and FBD | Wealth status, and residence |
| Restrepo-Méndez et al., 2015 | 13 Latin America and the Caribbean | Demographic and Health Surveys & Multiple Indicator Cluster Surveys: Bolivia (1998, 2008), Haiti (2000, 2012), Honduras (2005, 2011), Nicaragua (1997, 2001), Brazil (1996, 2006), Guatemala (1998, 2008), Peru (1996, 2012), Belize (2011), Colombia (1995, 2010), Costa Rica (2011), Suriname (2006, 2010), Dominican Republic (1996, 2007), Guyana (2006, 2009) | Equiplot | 4+ ANC visits, and SBA | Wealth status |
| Alkenbrack et al., 2015 | 74 LMICs | Demographic and Health Surveys: 1990-2013 | CI | ANC by SP, and FBD | Wealth status |

CI Concentration Index, CC Concentration curve, SII Slope index of inequality, RII Relative index of inequality, PAF Population attributable fraction, PAR Population attributable risk, BGV Between Group Variance, CV Coefficient of variation, SP Skilled provider, ANC Antenatal care, PNC Postnatal care, FBD Facility based delivery, SBA Skilled birth attendance, MHS Maternal healthcare services LIC low-income country, LwMIC lower middle-income country, UMIC upper middle-income country, LMIC low- and middle-income country
